# Psychosocial interventions for persons affected by Leprosy: A systematic review

**DOI:** 10.1371/journal.pmen.0000091

**Published:** 2024-08-09

**Authors:** Ann-Kristin Bonkass, Anil Fastenau, Sophie Stuetzle, Melanie Boeckmann, Mohammed Nadiruzzaman

**Affiliations:** 1 Department of Health, Ethics & Society, Care and Public Health Research Institute CAPHRI, Faculty of Health, Medicine and Life Sciences, Maastricht University, Maastricht, The Netherlands; 2 German Leprosy and Tuberculosis Relief Association (DAHW), Wuerzburg, Germany; 3 Heidelberg Institute of Global Health, University of Heidelberg, Germany; 4 Department of Global Health, Institute of Public Health and Nursing Research, University of Bremen, Germany; 5 Marie Adelaide Leprosy Center, Karachi, Pakistan; East China University of Science and Technology, CHINA

## Abstract

While multi-drug therapy revolutionised the treatment of physical symptoms for leprosy, a lack of psychosocial interventions, to combat the psychological burden of the disease, is noticeable. This is especially the case in a lower-middle-income country like India, where leprosy prevalence is highest, yet, it has one of the lowest rates of mental health services in place. This paper (i) conducts a systematic review to gather academic evidence on best practices of psychosocial care interventions of to leprosy patients from across the globe, and (ii) compiles good practices of mental wellbeing and quality of life to propose plausible actions for leprosy patients in India. Following the PRISMA protocol, keywords were searched in four databases, namely *PubMed*, *PsycInfo*, *Web of Science* and *Infolep*. After examining all 145 search results through inclusion and exclusion criteria, 17 peer reviewed research articles could qualify for final review exercise, whereby the data was systematically appraised. The systematic review reveals several successful psychosocial interventions implemented worldwide. These interventions were categorised into four sub-groups: educational, counselling, cognitive behavioural therapy, and technology-supported interventions. All the studies included in the analysis showcased effective psychosocial interventions that enhanced the quality of life and reduced depression, anxiety, and stress levels in individuals affected by leprosy. These findings highlighted several promising strategies that could be integrated into India’s mental healthcare system. The studies underscored the significance of involving healthcare professionals, and adopting innovative approaches. Consequently, this research proposes a comprehensive blend of diverse psychosocial interventions to alleviate the burden faced by leprosy-affected individuals in India. It is crucial to take into account various confounding factors and local contexts to tailor these interventions to the specific population group. Additionally, enhancing awareness and updating policies related to leprosy care are essential steps in reducing stigmatization against individuals with leprosy in India and other endemic regions.

## 1 Introduction

Leprosy, an enduring infectious malady attributed to Mycobacterium leprae, is historically intertwined with disability, societal bias, and discrimination. While the advent of multi-drug therapy (MDT) has revolutionized the management of leprosy’s physical manifestations and significantly reduced its global prevalence, leprosy remains a substantial contributor to the disease burden, with 133,802 documented cases in 2021 [1]. Nonetheless, the repercussions of leprosy are not confined to its physical symptoms; they frequently exert a profound influence on mental well-being and overall quality of life (QoL). Various factors converge to generate stigmatization of individuals afflicted by leprosy (PALs). Firstly, untreated individuals may develop conspicuous marks and disabilities owing to nerve damage. Secondly, a lack of public awareness fosters a hostile environment, nurturing both internalized and enacted stigma, as well as social exclusion [2]. Inability to perform daily tasks, engage in employment, or participate in community life can significantly compromise an individual’s well-being and QoL, curtailing their social inclusion. Barcelos et al. [3] underscore that PALs exhibit markedly lower QoL in comparison to individuals affected by other dermatological conditions.

Leprosy exerts a profound and far-reaching impact on various aspects of an individual’s life, consequently elevating the risk of developing mental comorbidities, specifically anxiety and depression. This pervasive influence extends into the social sphere, where individuals afflicted by the disease often grapple with stigma and resultant exclusion from their family and friends. Additionally, religious beliefs can compound this stigmatization, as leprosy is frequently misconstrued as a divine curse or retribution for one’s actions [4]. In low-resource settings, individuals confronting mental health challenges face a dual burden, navigating both their physical and psychological well-being, particularly in societies where mental health issues are considered taboo and are accompanied by social stigma [5]. Paradoxically, low-income countries (LICs) bear the brunt of the highest prevalence of mental illnesses while simultaneously grappling with a scarcity of mental health services, aggravated by inadequate financial and political investment [5].

The chasm between the demand for mental health treatment and its provision remains a global challenge. The inadequacy of mental health resources is particularly conspicuous within the Indian healthcare system. The 2016 National Survey of Mental Health Resources, conducted by the Indian Ministry of Health and Family Welfare, revealed an acute scarcity of mental health professionals, with a meager 0.3 psychiatrists per 100,000 people, primarily concentrated in the Western and Southern regions of the country [6,7]. Regrettably, preventive programs remain conspicuously absent and fall far short of addressing the needs of the population.

Only a small fraction of the annual budget, 5.2%, is allocated to health services, with mental health receiving even less attention, accounting for less than 1% [6,8]. Leprosy care predominantly emphasizes the biomedical aspects of treatment, specifically addressing physical symptoms through Multidrug Therapy (MDT) and symptom management. Unfortunately, personal and psychological symptoms are often overlooked within the leprosy treatment framework [9]. This underscores the urgent need to address the deficiency in mental health services for leprosy patients. As a response, this systematic review serves as a foundational step in highlighting the necessity of incorporating psychosocial interventions into leprosy care within the Indian healthcare system, drawing from global best practices. Researchers worldwide have demonstrated the positive impact of diverse psychosocial interventions, characterized by reductions in anxiety and depression symptoms, alongside enhancements in social functioning and self-esteem. Consequently, the provision of psychosocial support and other community-based mental health services can offer both cost-effective and equitable access to care, ultimately leading to improved health and social outcomes [10,11].

Nonetheless, there remains a scarcity of conclusive evidence to effectively address and mitigate the burden of mental health disorders and treatment requirements among Persons Affected by Leprosy (PALs) in India. To mitigate mental illness and elevate the Quality of Life (QoL) among leprosy patients, it is imperative to analyse existing data and tailor treatment plans to the unique needs of individuals [10]. Given the current dearth of data, this paper aims to offer an enlightening overview of psychosocial interventions that have demonstrated effectiveness in enhancing mental well-being and QoL among PALs globally. Furthermore, this paper examines the impact of a leprosy diagnosis on the QoL and mental well-being of patients. The identification of interventions proven effective in other countries will be instrumental in the establishment of mental healthcare services for leprosy patients in India. Consequently, this systematic review endeavours to present a comprehensive synthesis of available studies that focus on effective psychosocial interventions for PALs, with the goal of contributing to the development of evidence-based mental health programs in India [12].

## 2 Methodology

### 2.1 Search strategy

The research is based on a literature search conducted in the following databases *Pubmed*, *Web of Science*, *Infolep*, *PsycInfo* and *Google scholar* by a single assessor. The latest literature search was on June 28^th^, 2023. Reports, published studies, reviews and reports were searched by applying a systematic search following a comprehensive search strategy (Table 1). Thereby, the assessor accessed the databases through the institutional VPN-client provided by Maastricht University. The search terms were applied in a Title (TI) and Abstract (AB) search in order to refine the search. With the usage of appropriate search terms, relevant literature was identified according to the PICOS-Scheme (see Table 2), and a full-text screening performed. Only English literature was reviewed; however, the author reviewed Portuguese studies, which have been translated, to widen the search. Due to the lack of available data on mental health issues in leprosy patients, no restriction to the publication period was set. The overall review follows the methodology of the PRISMA checklist (S1 PRISMA Checklist) [13].

**Table 1:** Search strategy.

Pubmed Search– 28.06.2023		
**Number**	**Search terms**	**Results**
**#1**	"leprosy"[MeSH Terms] OR "hansen disease"[Title/Abstract] OR "leprosy"[Title/Abstract]	27,218
**#2**	"depressive disorder"[MeSH Terms] OR "anxiety disorders"[MeSH Terms] OR "suicide, attempted"[MeSH Terms] OR "mental disorders"[MeSH Terms] OR "quality of life"[MeSH Terms] OR "depressi*"[Title/Abstract] OR "anxi*"[Title/Abstract] OR "mental disorder"[Title/Abstract] OR "suicide"[Title/Abstract] OR "quality of life"[Title/Abstract] OR "mental wellbeing"[Title/Abstract] OR "wellbeing"[Title/Abstract]	2,308,048
**#3**	"counseling"[MeSH Terms] OR "psychiatric rehabilitation"[MeSH Terms] OR "peer group"[MeSH Terms] OR "psychotherapy"[MeSH Terms] OR "psychology, educational"[MeSH Terms] OR "psychosocial"[Title/Abstract] OR "counseling"[Title/Abstract] OR "psychiatric rehabilitation"[Title/Abstract] OR "peer group"[Title/Abstract] OR "psychology educational"[Title/Abstract] OR "mental health services"[Title/Abstract] OR "mental health services"[MeSH Terms] OR "peer counsel*"[Title/Abstract] OR "behavioral therapy"[Title/Abstract] OR "cognitive therapy"[Title/Abstract] OR "psychological"[Title/Abstract]	876,482
**#4**	#1 AND #2 AND #3	72
Web of Science search– 28.06.2023		
**Number**	**Search terms**	**Results**
**#1**	((TS = (Leprosy)) OR TS = (Hansen Disease)	8,452
**#2**	(((((((TS = (mental health)) OR TS = (mental disorder)) OR TS = (depressi*)) OR TS = (anxi*)) OR TS = (suicide)) OR TS = (quality of life)) OR TS = (mental wellbeing)) OR TS = (wellbeing)	1,813,360
**#3**	((((((((TS = (counsel*)) OR TS = (psychiatric rehabilitation)) OR TS = (psychological rehabilitation)) OR TS = (peer group)) OR TS = (psychotherapy)) OR TS = (mental health services)) OR TS = (psychosocial)) OR TS = (cognitive therapy)) OR TS = (behavioral therapy)	573,072
**#4**	#1 AND #2 AND #3	64
PsycInfo– 28.06.2023		
**Number**	**Search terms**	**Results**
**#1**	TI leprosy OR TI hansen’s disease OR AB leprosy OR AB hansen disease	268
**#2**	TI mental health OR TI mental disorders OR TI depressi* OR TI anxi* OR TI suicide OR TI quality of life OR AB mental health OR AB mental disorders OR AB depressi* OR AB anxi* OR AB suicide OR AB quality of life OR TI mental wellbeing OR TI wellbeing OR AB mental wellbeing OR AB wellbeing	796,661
**#3**	TI counseling OR TI psychiatric rehabilitation OR TI psychotherapy OR TI peer group OR TI mental health services OR TI cognitive therapy OR TI behavioral therapy OR AB counseling OR AB psychiatric rehabilitation OR AB psychotherapy OR AB peer group OR AB mental health services OR AB cognitive therapy OR AB behavioral therapy	234,150
**#4**	#1 AND #2 AND #3	0
**#5**	#1 AND #3	5
Infolep– 06.06.2023		
**Number**	**Search terms**	**Results**
**#1**	Leprosy	29,732
**#2**	Mental Health	253
**#3**	Stigma	347
**#4**	#1 AND #2 AND #3	61

**Table 2:** PICOS-Scheme.

**P** opulation	Leprosy patients
**I** ntervention	Effective psychological intervention to improve mental health wellbeing and quality of life in leprosy patients
**C** omparator	No Intervention
**O** outcome and endpoints	Improved mental wellbeing and QoL in leprosy patients
**S** etting	Leprosy clinics and populations with a high burden of leprosy worldwide

### 2.2 Eligibility, inclusion and exclusion

Firstly, papers dealing with a psychosocial intervention that addresses the relationship between mental wellbeing and leprosy were included. Thereby, a variety of mental disorders were included as can be seen in the search terms. Secondly, the full text of included studies needed to be published in English and must be publicly accessible. Therefore, original research published as peer-reviewed articles and/or book chapters in edited volumes was assessed. The author put no restriction on the publication year of potential studies as there are only limited papers available that assess the link between leprosy and mental health.

On the other hand, there are a number of factors that disqualified studies from being considered for this thesis. While leprosy is one of many NTDs, papers that merely considered other NTDs than leprosy were not valid for this assessment. As this review is looking at effective interventions to promote the QoL and mental wellbeing of PALs, studies that did not show any effect after implementation of the interventions were excluded as they would not be useful in applying the intervention to the Indian context. Furthermore, grey literature, manuscripts and editorials are not acceptable, as well as non-peer reviewed articles as they do not provide validated information.

### 2.3 Data extraction and analysis

**Fig 1:**
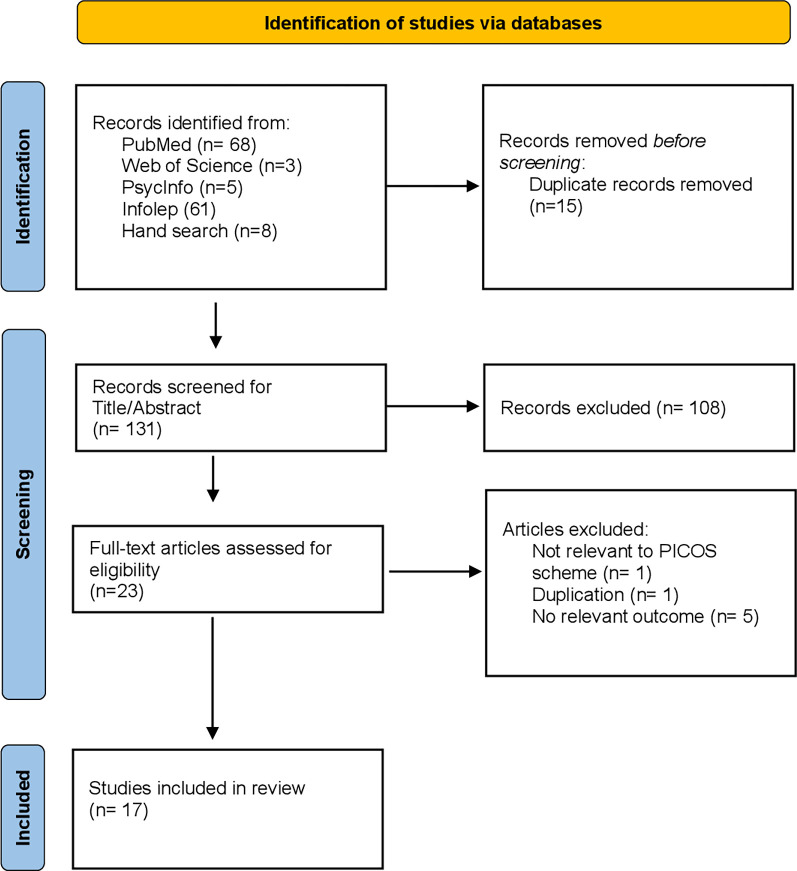
Flowchart of the included studies.

Overall, a total of 145 articles were found in the chosen databases through hand-searches in Google Scholar, based on the above-described systematic literature search. The systematic literature search identified 17 suitable studies that were included in this paper, according to the set inclusion and exclusion criteria. The data of the selected studies were extracted through Excel. A spreadsheet was developed assessing the title of the paper, author and publication year as well as how the papers answered the set research questions. An overview of the systematic literature search within the different databases can be found in Table 1. The flowchart in Fig 1 below illustrates the described literature search. Table 3 present the study characteristics of the selected studies in more detail. In order to assess the risk of bias of the included studies, the authors used the RoB RCTs tool [14] for randomized controlled trails, the NICE checklist [15] for qualitative studies, the MMAT tool [16] for mixed-method studies and finally, the critical review form [17] for quantitative studies. The extensive risk of bias analysis, which was performed in the beginning of the review to ensure low risk of bias, can be found in the Table 4. Thereby, the risk of bias within the studies was assessed as medium to low and suitable for the analysis of this review. By closely following the PRISMA guideline, which was developed beforehand, the authors attempted to adapt to the studies’ bias. During the assessment of the chosen studies, the author tried to ensure comparability and generalizability, which was limited due to heterogeneous study designs and interventions.

**Table 3:** Overview of the included interventions.

Psychoeducational interventions			
**Name/ Year and location of the study**	**Aim of the study + description**	**Used measures and tools**	**Outcome of the intervention**
**Mardhiyah (2019) [12]**	Three participants; 5 sessions à 90 minutes; aim: improve mental health of PALs through knowledge, mixed-methods design	Self-Reporting Questionnaire (SRQ)	Effective in two participants indicated by lower SRQ score after intervention
**Ahmed & Mohamed (2021) [19], Egypt**	72 participants; aim: improve psychological symptoms and self-care practices through educational intervention	structured interview questionnaire; patient’s self-care practices	After intervention: 27.8% average knowledge level and 61.1% good level regarding leprosy; Improved self-care practices; reduced psychological problems after intervention (p = 0.001)
Counseling interventions			
**Name/ Year and location of the study**	**Aim of the study + description**	**Used measures and tools**	**Outcome of the intervention**
**Lusli et al. (2016) [2], Brazil**	Aim: Rights-based counseling model; implementing human rights perspective into counseling sessions; individual, group or family sessions performed by 23 trained peer and lay counselors for 198 participants, mixed-methods design	Participation scale short (PSS); WHOQoL BREF; SARI scale (SSS)	SSS and PSS scales significantly decreased after intervention; Female gender found as a confounder; Participants felt more resilient after intervention
**Bhat et al. (2022) [20], India**	Aim: counseling interventions to improve the mental wellbeing of leprosy patients and to reduce stigma-related harm; 120 participants, mixed-methods design	Participation restriction scale	Gender (p = 0.002), age (p = 0.003), marital status (p = 0.017) and employment status (p = 0.0001) act as confounders; significant increase in participation of participants
**Floyd-Richard & Gurung (2000) [22], Nepal**	Aim: counseling intervention groups to improve psychological wellbeing; 117 participants, single sex and children groups; asked to remember three life phases: time before acquiring leprosy, after being infected with leprosy and the inpatient life at the hospital, qualitative research	Demographic data	Qualitative assessment, evaluated effective in reducing impact of stigmatization on PALs; weak outcome measures
**Dadun et al. (2016) [23], Indonesia**	237 PALs and two cohorts of community members (n = 213 and 375); additional socio-economic development with counseling sessions to improve psychological wellbeing, individual, group and family based, RCT design	Living and economic conditions; participation scale; WHOQoL BREF; SARI Stigma Scale	Intervention were effective in improving QoL (p = 0.013), participation restriction (p = 0.001), and stigma (p = 0.001) within intervention group
**Jay et al. (2021) [21]**	98 participants, aim: self-help groups to improve psychological wellbeing and participation restriction, quantitative research	Adjusted Likert scale to setting; SARI stigma scale; general health questionnaire; participation scale	Positive association between access to groups and reduced internalized stigma (p = 0.001); association between increased identification with self-help groups and improved psychological wellbeing (p = 0.003)
**Susanto et al. (2017) [24], Indonesia**	Aim: Experiences of PALs after joining self-care groups (SCG); 17 participants, qualitative research	Semi-structured interview questions	SCG supported participant’s ability to take care of themselves; improved QoL after intervention
**Van’t Noordende et al. (2021) [18], India**	80 participants, comprising 57 family members and 23 PALs; Aim: family-based counseling aimed at supporting participant’s protective abilities and resilience in two different areas Odisha and Telangana, mixed methods design	WHOQoL BREF; Connor-Davidson Resilience Scale (CD-RISC) for resilience level; semi-structured interviews	Increased QoL scores and higher CD-RISC score in Odisha after intervention, in Telangana CD-RICS only in PALs increased and QoL only increased for family members
**Jay et al. (2022) [25], Nepal**	98 participants, aim: counseling sessions to improve mental wellbeing and participation restriction, quantitative research	General Health Questionnaire	social belonging to a group has a positive effect on participant’s resilience (p = 0.01); better self- acceptance (p = 0.05); positive effect on people’s psychological wellbeing
Cognitive behaviour therapy			
**Name/ Year and location of the study**	**Aim of the study + description**	**Used measures and tools**	**Outcome of the intervention**
**Rahmawati & Yuniarti, 2017 [27],**	25 participants; aim: impact of CBT on level of depression in PALs, quantitative research	Beck Depression Inventory (BDI) questionnaire	Significant reduction in participant’s depression level (p = 0.001)
**Ramasamy et al., 2018 [26], India**	50 participants; Aim: evaluate the effectiveness of a progressive muscle relaxation technique (PMRT) on depression levels of leprosy patients, quantitative research	Self-developed questionnaire during face-to-face interviews	Significant difference between pre- and post-test scores for both depression and anxiety (both p = 0.001)
**Leite & Caldeira, 2015 [28], Brazil**	Aim: effectiveness of therapeutic workshops on patient’s QoL and depression level; 62 participants, quantitative research	WHOQoL BREF; BDI questionnaire	Workshops only effective to reduce level of moderate depression and no depressive symptoms at all
**Su et al., 2012 [29], Taiwan**	Aim: effectiveness of reminiscence group therapy in elderly leprosy patients; 129 participants divided in two groups, RCT design	Individual interviews, Geriatric Depression Scale–Short Form	Statistically significant reduction of depression level among elderly leprosy patients in intervention group
Technology-supported interventions / Other interventions			
**Name/ Year and location of the study**	**Aim of the study + description**	**Used measures and tools**	**Outcome of the intervention**
**Ramanathan et al., 1991 [31], India**	Aim: combination of counseling strategy with surgical correction of the physical symptoms of leprosy patients, quantitative research	Beck Depression Inventory (BDI) questionnaire; Taylor’s Manifest Anxiety Scale; Intelligence Scale	Anxiety and depression level differed significantly between pre and post intervention
**Dossa et al., 2021 [30], India**	34 participants; intervention and control group aims: conventional aerobic exercise programme paired with mental imagery for depression and anxiety, quantitative research	Depression, Anxiety and Stress Scale-21 (DASS-21)	Statistical significant reduction in depression (p = 0.0001), anxiety (p = 0.0002) and stress (p = 0.0001) in intervention group; comparing between control and intervention group only depression significant (0.0037)
**Geroge et al., 2013 [32], India**	Aim: focus on improved nursing intervention to reduce psychological symptoms in especially female leprosy patients who were admitted to a leprosy referral hospital; 40 participants, quantitative research	Hamilton Anxiety Rating Scale	Statistically significant reduction in anxiety score of female participants in all age groups (p = 0.01)

#### 1) RoB RCTs–Quality Assessment

Su, T.; Wu, L.; Lin, C.; The prevalence of dementia and depression in Taiwanese institutionalized leprosy patients, and the effectiveness evaluation of reminiscence therapy—a longitudinal, single‐blind, randomized control study.

#### Domain 1: Risk of bias arsing from the randomization process

**Table 4:** Quality Assessment of included studies.

Signalling questions	Comments	Response options (Yes/No/ No Information)
1.1 Was the allocation sequence random?	p. 188, Subjects: Purposive sampling	no
1.2 Was the allocation sequence concealed until participants were enrolled and assigned to interventions?	p. 189, Subjects: The remaining 102 participants were randomly assigned to one of the two study groups	yes
1.3 Did baseline differences between intervention groups suggest a problem with the randomization process?	p. 188; Subjects: Prospects were excluded from the study if they were too ill. Otherwise, both group members were form the same baseline group.	No
Risk of bias judgement	Randomization process was not fully achieved.	Some concerns

#### Domain 2: Risk of bias due to deviations from the intended interventions (effect of adhering to intervention)

**Table:** 

Signalling questions	Comments	Response options (Yes/No/No Information)
2.1. Were participants aware of their assigned intervention during the trial?	p. 189, Procedure: Participants were randomly assigned, however before the start of the study they were explained the purpose and procedure of the study.	Yes
2.2. Were carers and people delivering the interventions aware of participants’ assigned intervention during the trial?	p. 189, Procedure: the research team was trained beforehand for 3 months in order to prepare for the group division. Group A administered the intervention, while Group B was blind to the subject`s grouping./ p.195, Study Limitations: To avoid selection bias, the participants were simply labelled with serial numbers and were randomly assigned to masked blocks without prior stratification.	No
2.3. [If applicable:] If Y/PY/NI to 2.1 or 2.2: Were important non-protocol interventions balanced across intervention groups?	The distributions of the MMSE, CDR, and GDS‐SF scores of all the pre‐tested subjects are shown in Table 2 of the cited reference.	Yes
2.4. [If applicable:] Were there failures in implementing the intervention that could have affected the outcome?	p. 195, Study limitations: Researcher did not control participant’s medical treatment during the intervention period, which might have affected the prevalence of depression.	Yes
2.5. [If applicable:] Was there non- adherence to the assigned intervention regimen that could have affected participants’ outcomes?	Participants did adhere to the intervention.	No
2.6. If N/PN/NI to 2.3, or Y/PY/NI to 2.4 or 2.5: Was an appropriate analysis used to estimate the effect of adhering to the intervention?	p. 195, Results: There was an appropriate analysis as the decrease in depression was measured in the post-intervention groups.	Yes
Risk of bias judgement	Low RoB as there was only small deviation from the intended intervention.	Low/High/ some concerns

#### Domain 3: Missing outcome data

**Table:** 

Signalling questions	Comments	Response options
3.1 Were data for this outcome available for all, or nearly all, participants randomized?	p.195, Results: Outcomes were not randomized as the results were from control and intervention group.	No
3.2 If N/PN/NI to 3.1: Is there evidence that the result was not biased by missing outcome data?	p. 195, Study Limitations: Researchers only reported on avoiding selection bias, however, nothing was mentioned regarding missing data bias.	No Information
3.3 If N/PN to 3.2: Could missingness in the outcome depend on its true value?	n.a.	n.a.
3.4 If Y/PY/NI to 3.3: Is it likely that missingness in the outcome depended on its true value?	p.195, Study Limitations: Researchers did no mention missing data as a potential limitation of the study.	Partly no.
Risk-of-bias judgement	There has been some information that the researchers did not report upon which could be a factor for some concerns regarding the risk of bias.	Some concerns

#### Domain 4: Risk of Bias in measurement of the outcome

**Table:** 

Signalling questions	Comments	Response options
4.1 Was the method of measuring the outcome inappropriate?	p.195, Methodology consideration: The results of questionnaires (GDS‐SF and MMSE) may be biased by the interviewer’s prejudice according to the participants’ group assignment, and therefore, the interviewers in our study were blinded to the participants’ groups and were not involved in intervention in either the experimental or the control group.	No
4.2 Could measurement or ascertainment of the outcome have differed between intervention groups?	p.195, Study Limitations: Researchers attempted to neutralize the placebo effect of visiting by supportive interviewing of the control group, the attention–placebo effects in the experimental and control groups were still not equal, especially owing to the different visiting frequencies	Partly yes
4.3 If N/PN/NI to 4.1 and 4.2: Were outcome assessors aware of the intervention received by study participants?	p. 195, Methodology considerations: The results of questionnaires (GDS‐SF and MMSE) may be biased by the interviewer’s prejudice according to the participants’ group assignment, and therefore, the interviewers in our study were blinded to the participants’ groups and were not involved in intervention in either the experimental or the control group.	No
4.4 If Y/PY/NI to 4.3: Could assessment of the outcome have been influenced by knowledge of intervention received?	n.a.	n.a.
4.5 If Y/PY/NI to 4.4: Is it likely that assessment of the outcome was influenced by knowledge of intervention received?	n.a.	n.a.
Risk-of-bias judgement	The assessment for bias in the outcome measures was reported low, hence the assessor would judge a low risk of bias.	low

#### Domain 5: Risk of bias in selection of the reported result

**Table:** 

Signalling questions	Comments	Response options
5.1 Were the data that produced this result analysed in accordance with a pre-specified analysis plan that was finalized before unblinded outcome data were available for analysis?	n.a.	No Information
Is the numerical result being assessed likely to have been selected, on the basis of the results, from. . . 5.2.. . . multiple eligible outcome measurements (e.g. scales, definitions, time points) within the outcome domain?	n.a.	No Information
5.3. . . multiple eligible analyses of the data?	p.193f., Results: The outcome domains are presented in different tables looking at the different measurements.	Yes
Risk-of-bias judgement	Missing information regarding the reported results.	Some concerns
Overall Risk-of-bias judgement	Overall, the RCT has some information missing or has not reported upon them. Hence, the assessor rated the risk of bias as medium.	Some concerns

Study by: Dadun, D.; van Brakel, W.; Peters, R.; Lusli; M.; Zweekhorst, M.; Bunders; J.; Irwanto, Impact of socio-economic development, contact and peer counselling on stigma against persons affected by leprosy in Cirebon, Indonesia–a randomised controlled trial.

#### Domain 1: Risk of bias arsing from the randomization process

**Table:** 

Signalling questions	Comments	Response options (Yes/No/ No Information)
1.1 Was the allocation sequence random?	p.2, Abstract: pairs of the stigma reduction interventions were randomly allocated to sub-districts in Cirebon District, Indonesia./ p.7, Population and sample: To achieve adequate power for the quantitative assessment and the comparisons between the baseline and the end survey we calculated a required sample size of 600 people affected by leprosy (150 per study area) and 200 community members (50 per study area). The researchers used purposive sampling.	Partly no
1.2 Was the allocation sequence concealed until participants were enrolled and assigned to interventions?	No comments provided	No Information
1.3 Did baseline differences between intervention groups suggest a problem with the randomization process?	p. 7, Population and Sample: “There were two study populations: people affected by leprosy (under treatment or released from treatment up to 3 years earlier) and community members. We decided to follow people affected by leprosy over time (cohort) and to have two different cross-sectional observations of community members. To achieve adequate power for the quantitative assessment and the comparisons between the baseline and the end survey we calculated a required sample size of 600 people affected by leprosy (150 per study area) and 200 community members (50 per study area).” Hence, there were no differences anticipated.	No
Risk of bias judgement	Randomization process was not fully achieved.	Some concerns

#### Domain 2: Risk of bias due to deviations from the intended interventions (effect of adhering to intervention)

**Table:** 

Signalling questions	Comments	Response options (Yes/No/No Information)
2.1. Were participants aware of their assigned intervention during the trial?	No comments provided	No Information
2.2. Were carers and people delivering the interventions aware of participants’ assigned intervention during the trial?	p. 6f., Methods: the districts were allocated to be exploratory study areas however, the delivery people did know what group they were assigned to.	yes
2.3. [If applicable:] If Y/PY/NI to 2.1 or 2.2: Were important non-protocol interventions balanced across intervention groups?	No comments provided	No Information
2.4. [If applicable:] Were there failures in implementing the intervention that could have affected the outcome?	p. 19, Study limitations: In addition, despite best efforts of the research assistants to trace participants, we lost 22% to follow-up. This was higher than the anticipated 20%, again reducing power to detect smaller changes. The counselling and contact interventions were not structurally embedded in or linked to existing health or social services, in contrast to the SED intervention.	Yes
2.5. [If applicable:] Was there non- adherence to the assigned intervention regimen that could have affected participants’ outcomes?	No comments are provided however, the intervention in question is a counselling intervention. People were lost to follow-up.	No Information
2.6. If N/PN/NI to 2.3, or Y/PY/NI to 2.4 or 2.5: Was an appropriate analysis used to estimate the effect of adhering to the intervention?	n.a.	n.a.
Risk of bias judgement	Due to missing information about the intervention procedure as well as the non-blinding of the interviewers there is some concern about risk of bias.	some concerns

#### Domain 3: Missing outcome data

**Table:** 

Signalling questions	Comments	Response options
3.1 Were data for this outcome available for all, or nearly all, participants randomized?	No comments about the randomization process was provided.	No Information
3.2 If N/PN/NI to 3.1: Is there evidence that the result was not biased by missing outcome data?	p.19, Study limitations: n, despite best efforts of the research assistants to trace participants, we lost 22% to follow-up. This was higher than the anticipated 20%, again reducing power to detect smaller changes. Hence, the researchers considered the potential bias source.	Yes
3.3 If N/PN to 3.2: Could missingness in the outcome depend on its true value?	n.a.	n.a.
3.4 If Y/PY/NI to 3.3: Is it likely that missingness in the outcome depended on its true value?	n.a.	n.a.
Risk-of-bias judgement	Due to the missing information about the randomization process the risk of bias for this domain is rated as high.	High

#### Domain 4: Risk of Bias in measurement of the outcome

**Table:** 

Signalling questions	Comments	Response options
4.1 Was the method of measuring the outcome inappropriate?	p.7, Scales: A variety of quantitative and qualitative methods has been applied to assess stigma or proxies of stigma in people affected by leprosy and community members. The focus of this article is the quantitative assessment. Table 2 of the cited reference provides an overview of the five scales applied in this study. In addition to the scales, demographic information was collected, such as, age, sex, marital status, education, profession, household income, and disability grade.	No
4.2 Could measurement or ascertainment of the outcome have differed between intervention groups?	p.19, Study Limitations: However, the design of paired implementation and the reduced size of the eventual intervention cohort meant that there was insufficient power to detect smaller differences between the interventions.	Partly yes
4.3 If N/PN/NI to 4.1 and 4.2: Were outcome assessors aware of the intervention received by study participants?	As the study is written in the ‘we’-form, it is being assumed that the assessors knew the intervention that was admitted.	Yes
4.4 If Y/PY/NI to 4.3: Could assessment of the outcome have been influenced by knowledge of intervention received?	This bias was not reported upon.	No Information
4.5 If Y/PY/NI to 4.4: Is it likely that assessment of the outcome was influenced by knowledge of intervention received?	n.a.	n.a.
Risk-of-bias judgement	Due to missing or insufficient information in the study regarding the outcome measures, the risk of bias was rated as medium	Some concerns

#### Domain 5: Risk of bias in selection of the reported result

**Table:** 

Signalling questions	Comments	Response options
5.1 Were the data that produced this result analysed in accordance with a pre-specified analysis plan that was finalized before unblinded outcome data were available for analysis?	p. 7, Methods: The SARI project ran from 2010 till 2015. The baseline study was executed in 2011, the interventions were implemented in 2012 and 2013 and the final survey was executed in 2014.	yes
Is the numerical result being assessed likely to have been selected, on the basis of the results, from. . . 5.2.. . . multiple eligible outcome measurements (e.g. scales, definitions, time points) within the outcome domain?	p.8ff., Results: The Results are based on the provision of multiple eligible outcome measures as can be seen in the results part.	Yes
5.3. . . multiple eligible analyses of the data?	p.6, Methods: The analyses were set beforehand which means that there has been no additional analyses.	No
Risk-of-bias judgement	The results were presented in an appropriate manner wherefore the risk of bias is rated as low.	low
Overall Risk-of-bias judgement	Overall, the RCT has some information missing or has not reported upon them. Hence, the assessor rated the risk of bias as medium to high.	Some concerns

#### 2) MMAT Tool

**Table:** 

**Study by Bhat, L.; Khan, N.; Vaida, N.; Hassan, I.; Banday M. T.—A field study of counselling for improving social participation of leprosy affected persons in Jammu and Kashmir, 2022, India**				
Category of study design	Methodological quality criteria	Responses		
		**Yes**	**No**	**Can´t tell**
Screening questions (for all types)	S1: Are there clear research questions?	X		
	**Comments**: Research aim, p. 63, Objectives: to measure the correlation of various sociodemographic factors influencing participation restriction of leprosy-affected persons and the impact of interventions through counselling for preventing social exclusion.			
	S2: Do the collected data allow to address the research question?	X		
	**Comments**: p.64, Abstract: Counselling not only has an overall positive impact on social participation but its impact was highly influenced by demographic variables, particularly amongst those who are disabled or unemployed. / Conclusion, p. 75: The findings show that participation restriction was experienced by every participant and was significantly reduced to lower levels through counselling interventions for every grade of participation restriction.			
1.Qualitative	1.1 Is the qualitative approach appropriate to answer the research question?	X		
	**Comments**: p.66, Materials and Methods: Leprosy patients were interviewed without external interference in a private environment. Five sessions of group counselling with a group size of 20 leprosy-affected persons were conducted by a counsellor, a psychologist and two peer-counsellors chosen from previous counselling groups.			
	1.2 Are the qualitative data collection methods adequate to address the research question?	X		
	**Comments**: p.66, Materials and Methods: Accordingly, counselling was provided to help identify opportunities for work within and outside the home, to improve social participation by visiting places of worship, and to participate in social gatherings within and outside the community.			
	1.3 Are the findings adequately derived from the data?	X		
	**Comments**: p.72, Results: Direct quotes are provided from the counselling sessions. / p. 74, Discussion: This study found that a notable reduction in participation restriction by intervention through counselling.			
	1.4 Is the interpretation of results sufficiently substantiated by data	X		
	**Comments**: p. 72f., Results: Findings are supported by qualitative data derived from the counselling sessions and interviews, again also supported by quotes from the scripts.			
	1.5 Is there coherence between qualitative data sources, collection, analysis and interpretation?	X		
	**Comments**: p. 68, Measures: Table with detailed items of the Participation-Scale that has been assessed through the counselling and interview sessions.			
2. Quantitative randomized controlled trails	2.1 Is randomization appropriately performed?	n.a.		
	2.2. Are the groups comparable at baseline?	n.a		
	2.3. Are there complete outcome data?	n.a.		
	2.4. Are outcome assessors blinded to the intervention provided?	n.a.		
	2.5 Did the participants adhere to the assigned intervention?	n.a.		
3. Quantitative non-randomized	3.1 Are the participants representative of the target population?	n.a.		
	3.2. Are measurements appropriate regarding both the outcome and intervention (or exposure)?	n.a.		
	3.3. Are there complete outcome data?	n.a.		
	3.4. Are the confounders accounted for in the design and analysis?	n.a.		
	3.5. During the study period, is the intervention administered (or exposure occurred) as intended?	n.a.		
4. Quantitative descriptive	4.1. Is the sampling strategy relevant to address the research question?	X		
	**Comments**: p. 68, Results: A total of 185 leprosy patients living in different districts of Jammu and Kashmir were identified and contacted for this study. These were patients residing in leprosy colonies at Srinagar and Jammu, leprosy hospitals in various districts and patients residing at their native homes.			
	4.2. Is the sample representative of the target population?		X	
	**Comments**: p. 76, Limitations: Although the study covers one-fifth of the entire population of leprosy-affected persons residing in the region, all participants were residents of leprosy colonies as the study could not include leprosy-affected persons residing at their native places.			
	4.3. Are the measurements appropriate?	X		
	**Comments**: p.67, Measures: Sociodemographic data of the included leprosy patients were recorded to support findings. The P-scale is a semi-structured questionnaire to measure the extent to which people are restricted in social participation, suitable to be administered by nonprofessional interviewers.			
	4.4. Is the risk of nonresponse bias low?		X	
	**Comments**: p. 76, Limitations: Although the study covers one-fifth of the entire population of leprosy-affected persons residing in the region, all participants were residents of leprosy colonies as the study could not include leprosy-affected persons residing at their native places.			
	4.5. Is the statistical analysis appropriate to answer the research question?	X		
	Comments: p. 68, Statistical analysis: SPSS software package was used for data analysis, the distribution examined with Kolmogorov–Smirnov test and summarised as means and standard deviations for continuous variables with normal distribution. To examine the correlation between participation scores obtained from P-scale and sociodemographic parameters, Pearson’s correlation analysis and point biserial correlation was used.			
5. Mixed Methods	5.1. Is there an adequate rationale for using a mixed methods design to address the research question?	X		
	**Comments**: p. 66, Procedure: The study used quantitative methods to access the extent of participation restriction among leprosy-affected people and the role of counselling to improve their social participation. Leprosy patients were interviewed without external interference in a private environment.			
	5.2. Are the different components of the study effectively integrated to answer the research question?	X		
	**Comments**: p.68, Results: Data analysis for survey data and qualitative data; Statistically comparison of severity of participation restriction in terms of the different sociodemographic features.			
	5.3. Are the outputs of the integration of qualitative and quantitative components adequately interpreted	X		
	**Comments**: Quantitative findings described on page 69ff., qualitative data adequately interpreted throughout the results part described on page 72f., both data types further evaluated in the discussion part p.74f.			
	5.4. Are divergences and inconsistencies between quantitative and qualitative results adequately addressed			X
	**Comments**: No comments provided.			
	5.5. Do the different components of the study adhere to the quality criteria of each tradition of the methods involved?	X		
	**Comments**: p. 66, Procedures: The different component followed the described analysis within the study.			
Overall score	QUALITATIVE = 5 yes score; QUANTITATIVE = 3 yes score; MIXED-METHODS = 4 yes score **≙** 80% Quality of the study			

3)

**Table:** 

**Study by Mardhiyah, S.A., Psychoeducational intervention for improving mental health of leprosy patients, 2019, Indonesia**				
Category of study design	Methodological quality criteria	Responses		
		**Yes**	**No**	**Can´t tell**
Screening questions (for all types)	S1: Are there clear research questions?	X		
	**Comments**: Research aim, p.71, Methods: this research aimed to assess the effect of psychoeducational intervention for improving mental health of leprosy patients.			
	S2: Do the collected data allow to address the research question?	X		
	**Comments**: p.71, Methods: These interventions will be therapeutic and educative (psychoeducation) through experiential learning, for leprosy patients who experienced very complex psychosocial problems and the implementation of interventions will be delivery by individually, as an effort to create a sense of security and open attitude from the research participants. The outcome evaluation, the researcher will measure the extent to which the intervention caused changes in the direction expected in the participant of the research. Assessment was conducted b			
1.Qualitative	1.1 Is the qualitative approach appropriate to answer the research question?	X		
	**Comments**: p.72, Methods: Whereas, qualitatively, this research uses thematic analysis that aims to look for patterns shaped based on the theme.			
	1.2 Are the qualitative data collection methods adequate to address the research question?	X		
	**Comments**: p.73, methods: In this research, the researcher will quantification a particular psychological symptom and obtains a number of numbers that represent the symptoms, and also describe the symptoms with the qualitative approach so the information obtained will be broadly covered. The approach used in evaluating the effectiveness of intervention success in this research is based on how the intervention helps participants to change toward positive direction based on the psychological distress symptoms shown in them.			
	1.3 Are the findings adequately derived from the data?	X		
	**Comments**: p.77, Results: Total collected data from pre and post psychoeducational intervention consists of data from SRQ-20 questionnaire and testing of knowledge results.			
	1.4 Is the interpretation of results sufficiently substantiated by data	X		
	**Comments**: p.79, Results: Table 2 of the cited reference, Stage of Experiential Learning at the Implementation of Psychoeducation Interventions and Table 5, presentation of the Psychological Distress Symptoms of Participants derived from the qualitative data collection.			
	1.5 Is there coherence between qualitative data sources, collection, analysis and interpretation?	X		
	**Comments**: p. 75, Methods: Psychoeducational intervention is provided through the description, discussion, sharing, chores and direct exercise through experiential learning approach.			
2. Quantitative randomized controlled trails	2.1 Is randomization appropriately performed?	n.a.		
	2.2. Are the groups comparable at baseline?	n.a		
	2.3. Are there complete outcome data?	n.a.		
	2.4. Are outcome assessors blinded to the intervention provided?	n.a.		
	2.5 Did the participants adhere to the assigned intervention?	n.a.		
3. Quantitative non-randomized	3.1 Are the participants representative of the target population?	n.a.		
	3.2. Are measurements appropriate regarding both the outcome and intervention (or exposure)?	n.a.		
	3.3. Are there complete outcome data?	n.a.		
	3.4. Are the confounders accounted for in the design and analysis?	n.a.		
	3.5. During the study period, is the intervention administered (or exposure occurred) as intended?	n.a.		
4. Quantitative descriptive	4.1. Is the sampling strategy relevant to address the research question?	X		
	**Comments**: p. 72, Methods: Based on the results from two times screening at the public health center in Jakarta, the researcher got five leprosy patients who met the criteria to be the participants of the research. However, only three participants were willing (with inform consent) to take part in the intervention activity, namely participant 1, participant 2, and participant 3, while 2 other participants were absent and refused to take part in the activities planned by the researcher.			
	4.2. Is the sample representative of the target population?		X	
	**Comments**: p. 72, Methods: only 3 participants are included in the study which makes the results not generalizable to the overall target population.			
	4.3. Are the measurements appropriate?	X		
	**Comments**: p.83, Discussion: The improvement of mental health in this research is marked by the declining of psychological distress symptoms shown in research participants after psychological intervention is given.			
	4.4. Is the risk of nonresponse bias low?		X	
	**Comments**: p. 72, Methods: Low participants number, so nonresponse bias might be high.			
	4.5. Is the statistical analysis appropriate to answer the research question?			X
	Comments: p.72, Methods: Only mentioned that quantitative analysis is used for simple calculation but no further analysis is mentioned.			
5. Mixed Methods	5.1. Is there an adequate rationale for using a mixed methods design to address the research question?	X		
	**Comments**: p. 72, Methods: This research uses mixed analysis method between quantitative and qualitative. Quantitative analysis is used for simple calculation, using the descriptive analysis method, which is a statistic procedure to summarize, organize, and simplify the data. Whereas, qualitatively, this research uses thematic analysis that aims to look for patterns shaped based on the theme. Todd et al. say that mixing methods (quantitative and qualitative) are approaches whose functions are complementary, more understandable, and easier to use in the context of natural psychology.			
	5.2. Are the different components of the study effectively integrated to answer the research question?	X		
	**Comments**: p.77, Results: Total collected data from pre and post psychoeducational intervention consists of data from SRQ-20 questionnaire and testing of knowledge results.			
	5.3. Are the outputs of the integration of qualitative and quantitative components adequately interpreted	X		
	**Comments**: p.77, Results: Total collected data from pre and post psychoeducational intervention consists of data from SRQ-20 questionnaire and testing of knowledge results. The results are show in Table 6.			
	5.4. Are divergences and inconsistencies between quantitative and qualitative results adequately addressed			X
	**Comments**: No comments provided.			
	5.5. Do the different components of the study adhere to the quality criteria of each tradition of the methods involved?	X		
	**Comments**: p. 77, Results: Total collected data from pre and post psychoeducational intervention consists of data from SRQ-20 questionnaire and testing of knowledge results. The results are show in Table 6.			
Overall score	QUALITATIVE = 5 score; QUANTITATIVE = 2 score; MIXED-METHODS = 4 score **≙** 73% Quality of the study

**Table:** 

Study by Lusli, M.; Peters, R.; van Brakel, W.; Zweekhorst, M.; Iancu, S.; Bunders, J.; Irwanto, Regeer, B.; The Impact of a Rights-Based Counselling Intervention to Reduce Stigma in People Affected by Leprosy in Indonesia				
Category of study design	Methodological quality criteria	Responses		
		**Yes**	**No**	**Can´t tell**
Screening questions (for all types)	S1: Are there clear research questions?	X		
	**Comments**: p. 3, Introduction: Based on an exploratory study that aimed to understand the characteristics of people affected by leprosy and the views of the community, a draft module was developed, and piloted with 62 clients.			
	S2: Do the collected data allow to address the research question?	X		
	**Comments**: p.3, Introduction: The approach imports human rights principles into the counselling sessions, especially the right to healthcare, to help empower the clients./ p.20, Discussion: In the literature, counselling is often identified as a promising stigma reduction approach and this study confirms this.			
1.Qualitative	1.1 Is the qualitative approach appropriate to answer the research question?	X		
	**Comments**: p.7, Methods: As many paired interviews as possible were conducted (same interviewee for baseline and final survey). The IDIs (in-depth interviews) aimed to gain insight in the extent of stigma in people affected by leprosy before and after the intervention.			
	1.2 Are the qualitative data collection methods adequate to address the research question?	X		
	**Comments**: p.7, Methods: We aimed for 80 interviews for the baseline and 25 for the final survey. As many paired interviews as possible were conducted (same interviewee for baseline and final survey). Finally, two types of notes were prepared. The Participant Reflection Notes (PRN) were written at the end of the counselling and aimed to identify the benefits of counselling for the clients.			
	1.3 Are the findings adequately derived from the data?	X		
	**Comments**: p.10, Results: The findings are adequately derived from the data and are presented in tables and quotes in the results chapter.			
	1.4 Is the interpretation of results sufficiently substantiated by data	X		
	**Comments**: p. 10, Results: Findings are supported by qualitative data derived from the interview sessions, which are added with quotes.			
	1.5 Is there coherence between qualitative data sources, collection, analysis and interpretation?	X		
	**Comments**: p. 20, Results: The findings show that integrating three types of counselling contributed to its effectiveness. The counselling skills and attitudes that the lay and peer counsellors needed for each type overlapped somewhat, but there were also important differences (e.g. facilitating a peer group versus individual counselling).			
2. Quantitative randomized controlled trails	2.1 Is randomization appropriately performed?	n.a.		
	2.2. Are the groups comparable at baseline?	n.a		
	2.3. Are there complete outcome data?	n.a.		
	2.4. Are outcome assessors blinded to the intervention provided?	n.a.		
	2.5 Did the participants adhere to the assigned intervention?	n.a.		
3. Quantitative non-randomized	3.1 Are the participants representative of the target population?	n.a.		
	3.2. Are measurements appropriate regarding both the outcome and intervention (or exposure)?	n.a.		
	3.3. Are there complete outcome data?	n.a.		
	3.4. Are the confounders accounted for in the design and analysis?	n.a.		
	3.5. During the study period, is the intervention administered (or exposure occurred) as intended?	n.a.		
4. Quantitative descriptive	4.1. Is the sampling strategy relevant to address the research question?	X		
	**Comments**: p. 6, Results: Three scales were used: the SARI Stigma Scale (SSS), Participation Scale Short (PSS) and the World Health Organization Quality of Life instrument (WHO-QOL BREF). The Participation scale assesses participation restrictions and is based on the Participation domain of the International Classification of Functioning, Disability and Health.			
	4.2. Is the sample representative of the target population?	X		
	**Comments**: p.1, Methodology: The sample size differs per method, for example, data regarding 67 counselling clients and 57 controls from a cohort, and notes from 207 counselling clients were examined./ p. 7, Research methods: Based on a sample-size calculation and an anticipated loss to follow-up it was decided that 600 people affected by leprosy had to be part of the quantitative part of the baseline.			
	4.3. Are the measurements appropriate?	X		
	**Comments**: p.4, Theoretical framework: We decided to assess experiences of stigma, and two aspects that are negatively affected by it: participation and quality of life. The effect of leprosy or leprosy related stigma on the quality of life is interesting but has been little studied, and when it has, the results are mixed.			
	4.4. Is the risk of nonresponse bias low?			X
	**Comments**: No information provided			
	4.5. Is the statistical analysis appropriate to answer the research question?	X		
	Comments: p. 7, Data management and data analysis: The quantitative data was entered into an Epi Info for Windows database (version 3.5.3) and analysed using Stata 12.1 by RP. Demographic variables included sex, age (in years), married (yes/no), education, disability grade (0/1/2).			
5. Mixed Methods	5.1. Is there an adequate rationale for using a mixed methods design to address the research question?	X		
	**Comments**: p. 1, Methodology: Mixed methods (e.g. three scales, interviews, focus group discussions and reflection notes) were used to assess the impact of the intervention, which ran over a two-year period./ p.6, Research methods: Mixed methods were used to get an in-depth understanding of the effects of the counselling and how these were achieved (see questions in introduction) and to still be able to generalize the findings.			
	5.2. Are the different components of the study effectively integrated to answer the research question?	X		
	**Comments**: p.7, Data management and data analyses: The interviews and FGDs were recorded, transcribed and translated into English. This data was analysed by ML. The quantitative data was entered into an Epi Info for Windows database (version 3.5.3) and analysed using Stata 12.1 by RP. Demographic variables included sex, age (in years), married (yes/no), education, disability grade (0/1/2).			
	5.3. Are the outputs of the integration of qualitative and quantitative components adequately interpreted	X		
	**Comments**: p. 9ff, Results: Detailed outputs of the analyses of the qualitative and quantitative data available in results part as tables and quotes from the interviews.			
	5.4. Are divergences and inconsistencies between quantitative and qualitative results adequately addressed			X
	**Comments**: No comments provided. However, the different methods are used complementary in order to support each other’s findings.			
	5.5. Do the different components of the study adhere to the quality criteria of each tradition of the methods involved?	X		
	**Comments**: p. 6, Research methods: The validity [of the included measurements] has been tested and was found to be adequate in an Indonesian speaking sample.			
Overall score	QUALITATIVE = 5 yes score; QUANTITATIVE = 4 yes score; MIXED-METHODS = 4 yes score **≙** 86,6% Quality of the study			

4)

**Table:** 

**Study by Van’t Noordende, A. T., Da Bakirtzief Silva Pereira, Z., Biswas, P., Ilyas, M., Krishnan, V., Parasa, J., Kuipers, P. (2021). Strengthening individual and family resilience against leprosy-related discrimination. A pilot intervention study. PLoS Neglected Tropical Diseases, 15(4), e0009329. https://doi.org/10.1371/journal.pntd.0009329**				
Category of study design	Methodological quality criteria	Responses		
		**Yes**	**No**	**Can´t tell**
Screening questions (for all types)	S1: Are there clear research questions?	X		
	**Comments**: Introduction, p. 3: The current study aimed to develop and pilot an intervention to strengthen individual and family resilience against leprosy-related discrimination.			
	S2: Do the collected data allow to address the research question?	X		
	**Comments**: p.3, Methods, Study design and study area: We used a quasi-experimental, before-after study design using mixed methods data collection./ p.4, Description of the intervention: The contents of the intervention were based on a recent scoping review, a qualitative exploration on sources of strength and resilience, determinants of individual, family and community resilience among families in low- and middle-income contexts and the family resilience framework.			
1.Qualitative	1.1 Is the qualitative approach appropriate to answer the research question?	X		
	**Comments**: p.4, Description of the intervention: In addition, our qualitative exploration on sources of strength and resilience identified the importance of supporting family and social relationships, providing accurate information about leprosy, and acknowledging spiritual beliefs. We therefore added a separate session about knowledge about leprosy, and also integrated these other concerns.			
	1.2 Are the qualitative data collection methods adequate to address the research question?	X		
	**Comments**: p. 6, Data analysis: Qualitative data (detailed notes of family responses to interview questions, as well as staff records of weekly meetings) were thematically analysed, identifying key elements of the intervention described as beneficial by participants./ p.9, Qualitative data: It was evident from the interview notes that participants greatly valued the 10-week program and enjoyed the practical, vignette- and story-based approach. Qualitative feedback indicated that participants enjoyed the social dimensions of the program. In feedback some noted that the project gave them more confidence to “develop relationship(s) with others” (AD:BS).			
	1.3 Are the findings adequately derived from the data?	X		
	**Comments**: p.10, Content of Intervention: We therefore considered knowledge a tool to help family members address and challenge misconceptions and reduce (community) stigma. […] Qualitative responses indicated that the social dimensions of the intervention were especially appreciated by the participants. Accordingly, there was a significant increase of scores in the domains “social relationships” and “environment” of the WHOQOL-BREF scale in both states post-intervention.			
	1.4 Is the interpretation of results sufficiently substantiated by data	X		
	**Comments**: p.11., Impact of Intervention to resilience: The intervention in the present study significantly improved resilience scores in participants from Odisha state post-intervention, but not in participants from Telangana state.[…] Qualitative responses indicated that the social dimensions of the intervention were especially appreciated by the participants.			
	1.5 Is there coherence between qualitative data sources, collection, analysis and interpretation?	X		
	**Comments**: p. 12, Conclusion: This pilot study showed that the 10-week family-based intervention to strengthen resilience among persons affected by leprosy and their family members is feasible, and has the potential to improve resilience and quality of life.			
2. Quantitative randomized controlled trails	2.1 Is randomization appropriately performed?	n.a.		
	2.2. Are the groups comparable at baseline?	n.a		
	2.3. Are there complete outcome data?	n.a.		
	2.4. Are outcome assessors blinded to the intervention provided?	n.a.		
	2.5 Did the participants adhere to the assigned intervention?	n.a.		
3. Quantitative non-randomized	3.1 Are the participants representative of the target population?	n.a.		
	3.2. Are measurements appropriate regarding both the outcome and intervention (or exposure)?	n.a.		
	3.3. Are there complete outcome data?	n.a.		
	3.4. Are the confounders accounted for in the design and analysis?	n.a.		
	3.5. During the study period, is the intervention administered (or exposure occurred) as intended?	n.a.		
4. Quantitative descriptive	4.1. Is the sampling strategy relevant to address the research question?	X		
	**Comments**: p. 4, Participants and sampling procedure: Persons who had been treated for leprosy (‘persons affected’) and their family members were included in the study. Participants were purposively sampled; contacted through the networks of Lepra Society (Odisha cohort) and Hyderabad Leprosy Control & Health Society (HLCHS) (Telangana cohort)./ p.1, Findings: Eighty participants across 20 families were included in the study (23 persons affected and 57 family members).			
	4.2. Is the sample representative of the target population?	X		
	**Comments**: p. 4, Participants and sampling procedure: According to Hertzog, if the aim of the pilot study is to demonstrate intervention efficacy in a single group, a sample size of 20–25 is adequate. A sample size of 10–20 participants per group is adequate when trying to determine group differences. Since this was a proof of concept (pilot) study in which we wanted to explore outcomes of a trial intervention, but also wanted to determine if there are any differences between rural and urban areas, we aimed to include families from rural as well as urban areas.			
	4.3. Are the measurements appropriate?	X		
	**Comments**: p. 10, Discussion: Findings from this study suggest that a 10-week family-based intervention to strengthen resilience among persons affected by leprosy and their family members is feasible and has the potential to improve resilience and quality of life.			
	4.4. Is the risk of nonresponse bias low?			X
	**Comments**: No comments provided.			
	4.5. Is the statistical analysis appropriate to answer the research question?	X		
	Comments: p. 7f., Results: CD-RISC and WHOQOL-BREF test were taken before and after intervention, with usage of Wilcoxon signed-rank test and p-test./ p. 6, Data analysis: Quantitative data were collected on paper forms and entered in a database created using Epi Info. The data were analysed using IBM SPSS Statistics 24. Differences between participants from Odisha and Telangana state were evaluated using the Mann-Whitney U test for continuous variables, X2 statistics for categorical variables and Fisher’s Exact test for categorical variables for which the expected values in one of the cells of the contingency table was less than five.			
5. Mixed Methods	5.1. Is there an adequate rationale for using a mixed methods design to address the research question?	X		
	**Comments**: p. 1, Abstract: We used a quasi-experimental, before-after study design with a mixed methods approach.			
	5.2. Are the different components of the study effectively integrated to answer the research question?	X		
	**Comments**: p.4, Description if Intervention: The intervention was designed to strengthen the resilience of families struggling with leprosy-related discrimination, by strengthening their protective abilities and capacity to overcome adversity. The contents of the intervention were based on a recent scoping review, a qualitative exploration on sources of strength and resilience, determinants of individual, family and community resilience among families in low- and middle-income contexts and the family resilience framework.			
	5.3. Are the outputs of the integration of qualitative and quantitative components adequately interpreted	X		
	**Comments**: p.5, Discussion: We used two validated questionnaires: the Connor-Davidson Resilience Scale (CD-RISC) and WHOQOL-BREF. In addition, semi-structured interviews to assess participant satisfaction were conducted with each family post-intervention, with facilitators taking notes of the responses of participants to each question./ p.6, Data analysis: Quantitative data were collected on paper forms and entered in a database created using Epi Info. The data were analysed using IBM SPSS Statistics 24.			
	5.4. Are divergences and inconsistencies between quantitative and qualitative results adequately addressed			X
	**Comments**: No comments provided.			
	5.5. Do the different components of the study adhere to the quality criteria of each tradition of the methods involved?	X		
	**Comments**: p. 3, Ethics statement: Written informed consent was obtained from all participants prior to data collection.			
Overall score	QUALITATIVE = 5 yes score; QUANTITATIVE = 4 yes score; MIXED-METHODS = 4 yes score **≙** 90% Quality of the study			

#### 3) Critical review form–Quantitative studies

**Table:** 

Citation	Ahmed, A. L., & Mohamed, N. A. (2021). Effect of Educational Program on Health Consequences of Patients with Leprosy. Evidence-Based Nursing Research, 3(3), 12. https://doi.org/10.47104/ebnrojs3.v3i3.209	
Study purpose: Was the purpose stated clearly? ☒Yes ☐No	*Outline the purpose of the study*. p. 36, Aim of the study: The present study aimed to evaluate the effect of educational program on the health consequences of patients with leprosy. *How does the study apply to your research question*? The study assesses how a psychosocial intervention positively impacts the health consequences, which included the mental wellbeing, of persons affected by leprosy.	
Literature Was relevant background literature reviewed? ☒Yes ☐No	*Describe the justification of the need for this study*: p.36, Introduction: Leprosy is a matter of public health and public education; it is everyone’s obligation to at least know that it is out there to break out prejudice and stigma. Nurses have an influential and educational role in society. They are among those who come in close contact with the health-related stigma, enhancing patients’ awareness and participating in development planning activities to reduce stigma and discrimination against persons affected by leprosy	
Design: ☐Randomized (RCT) ☐Cohort ☐Single case design ☒before and after ☐case-control ☐cross-sectional ☐case study	*Describe the study design*. *Was the design appropriate for the study question*? *(e*.*g*., *for knowledge level about this issue*, *outcomes*, *ethical issues*, *etc*.): p.36, Research Design: quasi-experimental (pre/post-test) design was used; It was utilized to compare between study group pre/post-education program. *Specify any biases that may have been operating and the direction of their influence on the results*: No biases were reported upon.	
Sample N = 72 Was the sample described in detail? ☒Yes ☐No Was sample size justified? ☒Yes ☐No ☐N/A	*Sampling (who; characteristics; how many; how was sampling done*?*) If more than one group*, *was there similarity between the groups*?: Purposive sample of 72 participants, inclusion criteria explained. *Describe ethics procedures*. *Was informed consent obtained*?: p. 38, Procedures: Before data collection, all approval letters were obtained to conduct the study after explaining the purpose of the study. Official permission was obtained through an issued letter from the Dean of Faculty of Nursing, Kafr Elsheikh University, to the Director of Dermatology Hospital to conduct this study and obtain permission to collect the research data. Then, the patient’s verbal agreement to contribute to this study was obtained after clarification of the purpose of the study. The researchers initially introduced themselves to all patients.	
Outcomes Were the outcome measures reliable? ☒Yes ☐No ☐Not addressed Were the outcome measures valid? ☒Yes ☐No ☐Not addressed	*Specify the frequency of outcome measurement (i*.*e*., *pre*, *post*, *follow-up)*: p. 38, Procedures: pilot study was conducted but data was not included; By using a pre-testing tool to assess the patient’s physical problems and needs, as well as to identify patient’s demographic data, knowledge, attitude, practices, as reported by patients suffering from leprosy.	
	*Outcome areas*: SES characteristics Medical history of patients Knowledge level of leprosy patients	*List measures used*: Structured Interviewing questionnaire Health Consequences Assessment Questionnaire Health Consequences Assessment Questionnaire
Intervention Intervention was described in detail? ☒Yes ☐No ☐Not addressed Contamination was avoided? ☐Yes ☐No ☒Not addressed ☐N/A Cointervention was avoided? ☒Yes ☐No ☐Not addressed ☐N/A	*Provide a short description of the intervention (focus*, *who delivered it*, *how often*, *setting)*. *Could the intervention be replicated in practice*? The educational program construction included 4 phases (preparatory, assessment, planning/implementation and evaluation phase). The researchers developed a structured interviewing questionnaire on the basis of current literature including the following items: Meaning of disease, mode of transmission, methods of leprosy infection, source of leprosy infection, symptoms, complications, treatment, and laboratory tests. Due to the close description of what the questionnaire included and how the intervention was performed, the intervention is likely to be replicated in practice.
Results Results were reported in terms of statistical significance? ☒Yes ☐No ☐N/A ☐Not addressed Were the analysis method(s) appropriate? ☒Yes ☐No ☐Not addressed Clinical importance was reported? ☒Yes ☐No ☐Not addressed		*What were the results*? *Were they statistically significant (i*.*e*., *p < 0*.*05)*? *If not statistically significant*, *was study big enough to show an important difference if it should occur*? *If there were multiple outcomes*, *was that taken into account for the statistical analysis*? p.39, Results: As shown in Table 7, there is a statically significant relation between total knowledge, practice, and attitude in pre and post-program phases (p<0.001). Table 8 clarifies a statistically significant difference between pre and post-program regarding total knowledge, practice, and attitude mean scores. The comparison between the mean and standard deviation of knowledge level, practice level, and attitude level pre/post-educational program was significant (p<0.001).
Drop-outs were reported? ☐Yes ☒No	*Did any participants drop out from the study*? *Why*? *(Were reasons given and were drop-outs handled appropriately*?) No dropouts were reported.	
Conclusion and Implications Conclusions were appropriate given study methods and results ☒Yes ☐No	*What did the study conclude*? *What are the implications of these results for practice*? *What were the main limitations or biases in the study*? Educational programs greatly improved patients’ knowledge, practice level, patient attitude, and health needs with leprosy as physiological needs, psychological needs, social needs. Recommendations were based on the findings and include that health education programs should be conducted for the public, too, to increase attitude towards patients with leprosy.

**Table:** 

Citation	Leite, S. C. C., & Caldeira, A. P. (2015). Therapeutic workshops and psychosocial rehabilitation for institutionalised leprosy patients. *Ciencia & Saude Coletiva*, *20*(6), 1835–1842. https://doi.org/10.1590/1413-81232015206.16412014	
Study purpose: Was the purpose stated clearly? ☒Yes ☐No	*Outline the purpose of the study*. p. 1836, Introduction: This study aimed to evaluate the impact of a series of therapeutic workshops specially developed for this target audience by calculating and analysing depression and quality of life scores among a group of patients in the CSSFé before and after the implementation of activities. *How does the study apply to your research question*? The study assesses how therapeutical workshops positively impact the mental health of persons affected by leprosy.	
Literature Was relevant background literature reviewed? ☒Yes ☐No	*Describe the justification of the need for this study*: p. 1836, Introduction: Studies have shown that the use of therapeutic workshops in mental health care is a useful tool for promoting resocialisation and individual integration into groups, through activities that stimulate collective experiences and thinking based on a psychosocial approach and respect for individuality, alterity and subjectivity.	
Design: ☐Randomized (RCT) ☐Cohort ☐Single case design ☒before and after ☐case-control ☐cross-sectional ☐case study	*Describe the study design*. *Was the design appropriate for the study question*? *(e*.*g*., *for knowledge level about this issue*, *outcomes*, *ethical issues*, *etc*.): p.1836, Methods: The same assessment questionnaires were conducted with the patients before carrying out the workshops (March 2011) and six months after the completion of activities (October 2011).; The study complied with all ethical standards and was conducted with the previous approval of the managers of the CSSFé. All patients signed an informed consent form and the project was approved by the research ethics committees of the State University of Montes Claros and the Fundação Hospitalar do Estado de Minas Gerais–FHEMIG. *Specify any biases that may have been operating and the direction of their influence on the results*: p.1840, Discussion: small sample size, further response bias might has occurred as participants tended to provide responses which they believed would be pleasing to the interviewer as an expression of gratitude for their activities.	
Sample N = 62 Was the sample described in detail? ☒Yes ☐No Was sample size justified? ☒Yes ☐No ☐N/A	*Sampling (who; characteristics; how many; how was sampling done*?*) If more than one group*, *was there similarity between the groups*?: p. 1837, Methods: 62 participants were recruited for the study, The same assessment questionnaires were conducted with the patients before carrying out the workshops (March 2011) and six months after the completion of activities (October 2011). *Describe ethics procedures*. *Was informed consent obtained*?: p. 1837, Methods: The study complied with all ethical standards and was conducted with the previous approval of the managers of the CSSFé. All patients signed an informed consent form and the project was approved by the research ethics committees of the State University of Montes Claros and the Fundação Hospitalar do Estado de Minas Gerais–FHEMIG.	
Outcomes Were the outcome measures reliable? ☒Yes ☐No ☐Not addressed Were the outcome measures valid? ☒Yes ☐No ☐Not addressed	*Specify the frequency of outcome measurement (i*.*e*., *pre*, *post*, *follow-up)*:	
	*Outcome areas*: SES characteristics Symptoms of depression Participation restriction	*List measures used*: World Health Organization Quality of Life questionnaire (WHOQOL-BREF) 21-item Beck Depression Inventory Participation scale
Intervention Intervention was described in detail? ☒Yes ☐No ☐Not addressed Contamination was avoided? ☐Yes ☐No ☒Not addressed ☐N/A Cointervention was avoided? ☒Yes ☐No ☐Not addressed ☐N/A	*Provide a short description of the intervention (focus*, *who delivered it*, *how often*, *setting)*. *Could the intervention be replicated in practice*? P1837, Methods: The intervention was comprised of three different types of therapeutic workshops (music, arts and recreational games/activities) that were conducted on a weekly basis, with an approximate duration of two hours. The interventions were further held in female and male wards as well as in the communities for patients that tend to not leave their home.	
Results Results were reported in terms of statistical significance? ☒Yes ☐No ☐N/A ☐Not addressed Were the analysis method(s) appropriate? ☒Yes ☐No ☐Not addressed Clinical importance was reported? ☒Yes ☐No ☐Not addressed	*What were the results*? *Were they statistically significant (i*.*e*., *p < 0*.*05)*? *If not statistically significant*, *was study big enough to show an important difference if it should occur*? *If there were multiple outcomes*, *was that taken into account for the statistical analysis*? p. 1837, Methods: The Wilcoxon test for paired samples (“before” and “after”) was used to compare medians, while the chi-square test was used to compare proportions. The WHOQOL-BREF scores were computed using the software SPSS. p. 1837, Results: Significant changes were noticed for all domains except social relationships. There was a significant difference in the amount of patients that had to no symptoms of depression anymore (p = 0.001) as well as a significant reduction in patients with moderate depression (p = 0.001). Clinical importance: p. 1840, Discussion: This form of treatment may contribute towards improved psychosocial integrity, reduce self-stigma, and help patients to cope with stigma and prejudice, and, as a consequence, lead to a process of resocialisation and resignification of individual and collective histories.	
Drop-outs were reported? ☐Yes ☒No	*Did any participants drop out from the study*? *Why*? *(Were reasons given and were drop-outs handled appropriately*?) No information on patient’s dropout was provided.	
Conclusion and Implications Conclusions were appropriate given study methods and results ☒Yes ☐No	*What did the study conclude*? *What are the implications of these results for practice*? *What were the main limitations or biases in the study*? p. 1837, Discussion: The results show that there were positive changes in depression and quality of life scores after the implementation of the therapeutic workshops./ Therapeutic workshops have the potential to improve the quality of life as well as physical health, psychological health and environmental quality of life. In the case of severe depression there is the need for the complementary usage of anti-depressant.

**Table:** 

Citation	Alisha-Akbar-Dossa, Parag-Shrinivas-Ranade, & Rahul-Nagendrasingh-Bisen (2021). Effect of mental imagery on depression, anxiety and stress in instituitionalised leprosy patients–An experimental study. World Journal of Advanced Research and Reviews, 12(3), 296–303. https://doi.org/10.30574/wjarr.2021.12.3.0701	
Study purpose: Was the purpose stated clearly? ☒Yes ☐No	*Outline the purpose of the study*. p. 297, Aim: The study aimed to assess the effect of mental imagery on Depression, Anxiety and Stress in institutionalised Leprosy patients using the DASS-21 (Depression, Anxiety and Stress Scale -21). *How does the study apply to your research question*? The study assesses how mental imagery has a positive effect on the mental wellbeing of persons affected by leprosy.	
Literature Was relevant background literature reviewed? ☒Yes ☐No	*Describe the justification of the need for this study*: p. 297, Introduction: Mental Imagery (MI) can be referred to as the representations and the accompanying experience of sensory information without an actual external stimulus [12]. It plays a pivotal role in many mental disorders and clinicians can utilize it in the treatment of such disorders.	
Design: ☐Randomized (RCT) ☐Cohort ☐Single case design ☐before and after ☒case-control ☐cross-sectional ☐case study	*Describe the study design*. *Was the design appropriate for the study question*? *(e*.*g*., *for knowledge level about this issue*, *outcomes*, *ethical issues*, *etc*.): p. 297, Material and methods: This was a pre-requisite as the screening measure and outcome measure utilised in the study were both self-administered and provided in a language suitable to these patients. The Bett’s questionnaire was used as a screening tool to assess the imagery abilities of the patients. The DASS-21 (Depression, Anxiety and Stress Scale– 21) was administered at the beginning of the intervention and after 2 weeks. It has excellent Cronbach’s alpha values for reliability of 0.81, 0.89 and 0.78 for the subscales of depression, anxiety and stress respectively. *Specify any biases that may have been operating and the direction of their influence on the results*: p. 302, Limitations: The assessment of psychological parameters was based on the outcome measure, the DASS-21 which does not replace a face to face interview with a professional consultant for a clinical diagnosis of Depression, Anxiety or Stress disorders. p.297, Materials and Methods: Patients were also asked if they practice some other methods of relaxation such as Yoga, meditation etc or are on medications for depression or anxiety to account for factors which may confound the study. These patients were not excluded from the study on grounds of ethical considerations.	
Sample N = 34 Was the sample described in detail? ☒Yes ☐No Was sample size justified? ☒Yes ☐No ☐N/A	*Sampling (who; characteristics; how many; how was sampling done*?*) If more than one group*, *was there similarity between the groups*?: p. 297, Material and Methods: A total of 34 subjects residing in an institutionalised set-up in Pune participated in the study. The sample size was calculated based on the prevalence of psychiatric disorders occurring in these patients in India. The technique of sampling was convenient sampling and randomization of the subjects into two groups was done through a computer-generated random allocation chart. Only those patients who scored a minimum of 4 points on each item were recruited in the study (scores in the range of 35 to 140) which was the criteria for possessing basic imagery abilities. *Describe ethics procedures*. *Was informed consent obtained*?: p. 297, Material and Methods: Permission from the ethical committee of the institute was taken. Written informed consents from the participants were obtained. The subjects were screened according to the inclusion and exclusion criteria.	
Outcomes Were the outcome measures reliable? ☒Yes ☐No ☐Not addressed Were the outcome measures valid? ☒Yes ☐No ☐Not addressed	*Specify the frequency of outcome measurement (i*.*e*., *pre*, *post*, *follow-up)*:	
	*Outcome areas*: Presence of depression/ anxiety Demographic details	*List measures used*: DASS-21 (Depression, Anxiety and Stress Scale– 21) Initial screening
Intervention Intervention was described in detail? ☒Yes ☐No ☐Not addressed Contamination was avoided? ☐Yes ☐No ☒Not addressed ☐N/A Cointervention was avoided? ☐Yes ☐No ☒Not addressed ☐N/A	*Provide a short description of the intervention (focus*, *who delivered it*, *how often*, *setting)*. *Could the intervention be replicated in practice*? p. 297, Materials and methods: The experimental group received the conventional protocol with a mental imagery session lasting 10 minutes. The MI session was delivered via an audiotape with a pre-recorded voice. The treatment was delivered in the form of group session involving 5–7 people at a time, for both the control and experimental groups. Frequency of treatment for both the groups was four times per week for 2 weeks. A follow up through a diary and phone calls was maintained for the rest of the days.	
Results Results were reported in terms of statistical significance? ☒Yes ☐No ☐N/A ☐Not addressed Were the analysis method(s) appropriate? ☒Yes ☐No ☐Not addressed Clinical importance was reported? ☒Yes ☐No ☐Not addressed	*What were the results*? *Were they statistically significant (i*.*e*., *p < 0*.*05)*? *If not statistically significant*, *was study big enough to show an important difference if it should occur*? *If there were multiple outcomes*, *was that taken into account for the statistical analysis*? p.298, Results: There was a statistically significant difference between the conventional and experimental groups for depression (p = 0.0001), anxiety (p = 0.0002) and stress levels (p = 0.0001). The tables and graphs provided in the study display the statistically significant results. It can thus be summated that all the parameters of the DASS-21 (Depression, Anxiety and Stress) have shown statistically significant differences in the pre and post intervention scores in both the control and experimental groups with p values < 0.05 in each domain.	
Drop-outs were reported? ☒Yes ☐No	*Did any participants drop out from the study*? *Why*? *(Were reasons given and were drop-outs handled appropriately*?) p.300, Discussion: None of the patients dropped out of the study.	
Conclusion and Implications Conclusions were appropriate given study methods and results ☒Yes ☐No	*What did the study conclude*? *What are the implications of these results for practice*? *What were the main limitations or biases in the study*? p.301, Conclusion: According to the results obtained in the current study, mental imagery can be used as an effective adjunct to conventional aerobic exercises to reduce depression, anxiety and stress in the Leprosy affected population. Also, conventional aerobic exercises have a significant impact on the reduction of depression, anxiety and stress in this population. p. 302, Limitations: The assessment of psychological parameters was based on the outcome measure, the DASS-21 which does not replace a face to face interview with a professional consultant for a clinical diagnosis of Depression, Anxiety or Stress disorders.

**Table:** 

Citation	Jay, S., Winterburn, M., Jha, K., Sah, A. K., Choudhary, R., & Muldoon, O. T. (2022). A resilience building collaboration: A social identity empowerment approach to trauma management in leprosy-affected communities. Psychological Trauma: Theory, Research, Practice and Policy, 14(6), 940–947. https://doi.org/10.1037/tra0001160	
Study purpose: Was the purpose stated clearly? ☒Yes ☐No	*Outline the purpose of the study*. p.942, A Resilience-Building Reciprocal Collaboration: Our research work explicitly engages an empowerment paradigm, and our aim is to examine the extent to which a group-based initiative improves people’s own ability to manage, engage with, and adapt to their leprosy and associated social, economic, and health challenges. *How does the study apply to your research question*? The study assesses the strengths of a Social identity Empowerment Approach to promote the leprosy affected persons.	
Literature Was relevant background literature reviewed? ☒Yes ☐No	*Describe the justification of the need for this study*: p.940, Clinical Impact Statement: Built on the understanding that trauma and resilience are group-based phenomena, we present a group based social identity empowerment approach that orients to social position, resources, and power. We describe a reciprocal collaboration with the Nepal Leprosy Trust (NLT), a social development nongovernmental organization working with impoverished, stigmatized, and traumatized leprosy affected communities in rural Nepal. Results show that this group-based approach encourages shared social identity, which is the basis for learning, social participation, and psychological resilience.	
Design: ☐Randomized (RCT) ☐Cohort ☐Single case design ☒before and after ☐case-control ☐cross-sectional ☐case study	*Describe the study design*. *Was the design appropriate for the study question*? *(e*.*g*., *for knowledge level about this issue*, *outcomes*, *ethical issues*, *etc*.): p.943, Method: Data collection was longitudinal with two waves; the same participants completed measures at time 1 and again at time 2, six months later. p.944, Design and Approach to Analysis: To explore the value of participation in the self-help groups, we explored differences between Time 1 and Time 2 scores using paired samples t tests. *Specify any biases that may have been operating and the direction of their influence on the results*: Small sample size and no randomization of the participants.	
Sample N = 98 Was the sample described in detail? ☒Yes ☐No Was sample size justified? ☒Yes ☐No ☐N/A	*Sampling (who; characteristics; how many; how was sampling done*?*) If more than one group*, *was there similarity between the groups*?: p. 943, Participants: Time 1 sample N = 98; Time 2 sample N = 71; two months difference between the data collection; Participants were members of of 10 preexisting self-help groups in ten rural villages, in four districts (Dhanusha, Mahottari, Sarlahi, and Sindhuli) with high rates of leprosy and disability *Describe ethics procedures*. *Was informed consent obtained*?: p. 943, Procedure: Ethical approval was secured from our faculty research ethics committee. All procedures performed in study involving human participants were in accordance with the ethical standards of the institutional research committee and with the 1964 Helsinki declaration and its later amendments or comparable ethical standards. Informed consent was obtained from all individual participants included in the study.	
Outcomes Were the outcome measures reliable? ☒Yes ☐No ☐Not addressed Were the outcome measures valid? ☒Yes ☐No ☐Not addressed	*Specify the frequency of outcome measurement (i*.*e*., *pre*, *post*, *follow-up)*: p.943, Participants: Data collection was longitudinal with two waves; the same participants completed measures at time 1 and again at time 2, six months later.	
	*Outcome areas*: Resilience Self-Help group identification Perceived Access to Multiple Groups Health Literacy	*List measures used*: General Health Questionnaire Model adapted from Leach et al. Participation scale Adapted short assessment of health literacy
Intervention Intervention was described in detail? ☒Yes ☐No ☐Not addressed Contamination was avoided? ☐Yes ☐No ☒Not addressed ☐N/A Cointervention was avoided? ☐Yes ☐No ☒Not addressed ☐N/A	*Provide a short description of the intervention (focus*, *who delivered it*, *how often*, *setting)*. *Could the intervention be replicated in practice*? The researchers applied a social identity-based empowerment approach for ten self-help groups based in Nepal. This intervention is addresses the increase of knowledge and empowerment for patients affected by leprosy. The academic members of the team travelled to Nepal and ran training workshops with community outreach workers. To explore the value of participation in the self-help groups, we explored differences between Time 1 and Time 2 scores using paired samples t tests. this study extends previous work by examining whether the social curative resources afforded to people through the sense of group identification has the ability to assuage the adverse psychological trauma associated with a leprosy diagnosis	
Results Results were reported in terms of statistical significance? ☒Yes ☐No ☐N/A ☐Not addressed Were the analysis method(s) appropriate? ☒Yes ☐No ☐Not addressed Clinical importance was reported? ☒Yes ☐No ☐Not addressed	*What were the results*? *Were they statistically significant (i*.*e*., *p < 0*.*05)*? *If not statistically significant*, *was study big enough to show an important difference if it should occur*? *If there were multiple outcomes*, *was that taken into account for the statistical analysis*? p. 944, Results: The researchers found a statistically significant relationship of change in self-help group identification on change in resilience medicated by change in access to multiple groups (p = <0.05). Belonging in a self-help group was also an statistically significant factor regarding peer-to-peer learning about health (p = <0.05).	
Drop-outs were reported? ☒Yes ☐No	*Did any participants drop out from the study*? *Why*? *(Were reasons given and were drop-outs handled appropriately*?) p. 944, Procedure: The researchers reported on some missing data points as the participants were advised to answer only the questions, they were willing to answer. Only complete measures are included in the analysis.	
Conclusion and Implications Conclusions were appropriate given study methods and results ☒Yes ☐No	*What did the study conclude*? *What are the implications of these results for practice*? *What were the main limitations or biases in the study*? p.946, Discussion: The findings suggest that facilitating participation in the smallest of ways can have important consequences in terms of resilience. Self-help groups can make it easier for people to endure and enable participants’ social and psychological resources to cope with adversity.

**Table:** 

Citation	Jay, S., Winterburn, M., Choudhary, R., Jha, K., Sah, A. K., O’Connell, B. H., O’Donnell, A. T., Moynihan, A. B., & Muldoon, O. T. (2021). From social curse to social cure: A self‐help group community intervention for people affected by leprosy in Nepal. *Journal of Community & Applied Social Psychology*, *31*(3), 276–287. https://doi.org/10.1002/casp.2510	
Study purpose: Was the purpose stated clearly? ☒Yes ☐No	*Outline the purpose of the study*. p. 283, Discussion: The overarching aim of this research was to examine whether identifying with a community-based self-help group served as a social cure for people affected by leprosy. Specifically, we investigated if a self-help group intervention can establish a new supportive social identification, which can predict people’s ability to access multiple groups, reduced internalized stigma, and improved well-being. *How does the study apply to your research question*? The study assesses the impact of self-help group community intervention on the wellbeing of persons affected by leprosy.	
Literature Was relevant background literature reviewed? ☒Yes ☐No	*Describe the justification of the need for this study*: p. 277, Introduction: According to the Social Cure literature, new social identities developed in group-based interventions can play a powerful role in improving health. Typically, education and leadership training are provided with the goal of establishing a new valued social identity that will improve member’s access to multiple groups’ through social participation, and in this way decrease stigma, and increase well-being. The study makes an important contribution to social cure literature by investigating the role of social identification and access to multiple groups’ in stigma elimination in adverse circumstances. Additionally, it represents a break from an over-reliance in the social sciences on Western, Educated, Industrialized, Rich and Democratic (WEIRD) samples.	
Design: ☐Randomized (RCT) ☐Cohort ☐Single case design ☐before and after ☐case-control ☐cross-sectional ☐case study	*Describe the study design*. *Was the design appropriate for the study question*? *(e*.*g*., *for knowledge level about this issue*, *outcomes*, *ethical issues*, *etc*.): *Specify any biases that may have been operating and the direction of their influence on the results*:	
Sample N = Was the sample described in detail? ☐Yes ☐No Was sample size justified? ☐Yes ☐No ☐N/A	*Sampling (who; characteristics; how many; how was sampling done*?*) If more than one group*, *was there similarity between the groups*?: *Describe ethics procedures*. *Was informed consent obtained*?:	
Outcomes Were the outcome measures reliable? ☐Yes ☐No ☐Not addressed Were the outcome measures valid? ☐Yes ☐No ☐Not addressed	*Specify the frequency of outcome measurement (i*.*e*., *pre*, *post*, *follow-up)*:	
	*Outcome areas*:	*List measures used*:
Intervention Intervention was described in detail? ☐Yes ☐No ☐Not addressed Contamination was avoided? ☐Yes ☐No ☐Not addressed ☐N/A Cointervention was avoided? ☐Yes ☐No ☐Not addressed ☐N/A	*Provide a short description of the intervention (focus*, *who delivered it*, *how often*, *setting)*. *Could the intervention be replicated in practice*?	
Results Results were reported in terms of statistical significance? ☐Yes ☐No ☐N/A ☐Not addressed Were the analysis method(s) appropriate? ☐Yes ☐No ☐Not addressed Clinical importance was reported? ☐Yes ☐No ☐Not addressed	*What were the results*? *Were they statistically significant (i*.*e*., *p < 0*.*05)*? *If not statistically significant*, *was study big enough to show an important difference if it should occur*? *If there were multiple outcomes*, *was that taken into account for the statistical analysis*?	
Drop-outs were reported? ☐Yes ☐No	*Did any participants drop out from the study*? *Why*? *(Were reasons given and were drop-outs handled appropriately*?)	
Conclusion and Implications Conclusions were appropriate given study methods and results ☐Yes ☐No	*What did the study conclude*? *What are the implications of these results for practice*? *What were the main limitations or biases in the study*?

**Table:** 

Citation	**Rahmawati, I.; Yuniarti, E. V. (2017). The influence of cognitive behaviour therapy to decrease the level of depression for leprosy sufferer. International Journal of Nursing and Midwifery.**	
Study purpose: Was the purpose stated clearly? ☒Yes ☐No	*Outline the purpose of the study*. p. 69, Abstract: This present research is to reveal the influence of Cognitive Behaviour Therapy to decrease the level of depression for leprosy sufferer. *How does the study apply to your research question*? The study assesses how cognitive behaviour therapy can positively impact the level of depression of persons suffering from leprosy.	
Literature Was relevant background literature reviewed? ☒Yes ☐No	*Describe the justification of the need for this study*: p. 69, Abstract: One of non-pharmacology treatment that can decrease the level of depression is Cognitive Behaviour Therapy. Cognitive Behaviour Therapy, which is an approach with someone to recognize and to change the bad mindset or maladaptive. / p.40, Introduction: Cognitive Behavior Therapy is a new strategy to overcome problems and provide more lasting changes in fundamental attitudes and ways of behaving.	
Design: ☐Randomized (RCT) ☐Cohort ☐Single case design ☒before and after ☐case-control ☐cross-sectional ☐case study	*Describe the study design*. *Was the design appropriate for the study question*? *(e*.*g*., *for knowledge level about this issue*, *outcomes*, *ethical issues*, *etc*.): p.70, Research method: Design Pre-Experimental using the One-Group Pre-Post Test Design approach. The measuring tool uses a BDI Questionnaire (Beck Depression Inventory) which consists of 21 questions. *Specify any biases that may have been operating and the direction of their influence on the results*: No comments provided.	
Sample N = 25 Was the sample described in detail? ☒Yes ☐No Was sample size justified? ☐Yes ☐No ☒N/A	*Sampling (who; characteristics; how many; how was sampling done*?*) If more than one group*, *was there similarity between the groups*?: p. 70, Research methods: The population in this study there are all patients according to the criterion of the researcher. Sampling using nonprobability sampling technique that is consecutive sampling. The number of samples used in this study is part of leprosy patients is 25 respondents. Sociodemographic information about the participants is provided *Describe ethics procedures*. *Was informed consent obtained*?: No information on informed consent was provided. Participants were recruited according to the inclusion criteria set by the researchers.	
Outcomes Were the outcome measures reliable? ☒Yes ☐No ☐Not addressed Were the outcome measures valid? ☒Yes ☐No ☐Not addressed	*Specify the frequency of outcome measurement (i*.*e*., *pre*, *post*, *follow-up)*: Outcome measures were obtained before and after the implementation of the intervention.	
	*Outcome areas*: SES characteristics Depression Level	*List measures used*: BDI Questionnaire (Beck Depression Inventory) BDI Questionnaire (Beck Depression Inventory)
Intervention Intervention was described in detail? ☒Yes ☐No ☐Not addressed Contamination was avoided? ☐Yes ☐No ☒Not addressed ☐N/A Cointervention was avoided? ☐Yes ☐No ☒Not addressed ☐N/A	*Provide a short description of the intervention (focus*, *who delivered it*, *how often*, *setting)*. *Could the intervention be replicated in practice*? By implementing cognitive behaviour therapy, the patients are taught to face problems and obstacles by applying this new approach. Thereby, the study participants’ depression level was assessed before and after the intervention whereby one was able to see the positive impact of cognitive behaviour therapy on the mental wellbeing of persons affected by leprosy.	
Results Results were reported in terms of statistical significance? ☒Yes ☐No ☐N/A ☐Not addressed Were the analysis method(s) appropriate? ☒Yes ☐No ☐Not addressed Clinical importance was reported? ☒Yes ☐No ☐Not addressed	*What were the results*? *Were they statistically significant (i*.*e*., *p < 0*.*05)*? *If not statistically significant*, *was study big enough to show an important difference if it should occur*? *If there were multiple outcomes*, *was that taken into account for the statistical analysis*? p.71, Results: The researchers reported a statistically significant reduction in depression level of leprosy patients after the intervention. / p.73, Conclusion: The Influence of Cognitive Behaviour Therapy Against Decreasing Depression Rate with statistical test result showed p-value (0.001). H0 is rejected, so there is Influence of Cognitive Behavior Therapy Against Decreasing Depression Rate In Leprosy Patients in Inpatient Room Leprosy Leprosy Hospital Sumberglagah Pacet–Mojokerto.	
Drop-outs were reported? ☐Yes ☒No	*Did any participants drop out from the study*? *Why*? *(Were reasons given and were drop-outs handled appropriately*?) No information was provided.	
Conclusion and Implications Conclusions were appropriate given study methods and results ☒Yes ☐No	*What did the study conclude*? *What are the implications of these results for practice*? *What were the main limitations or biases in the study*? p.73, Conclusion: The Influence of Cognitive Behavior Therapy Against Decreasing Depression Rate with statistical test result showed p-value (0.001). p.73, Suggestions: Health workers should also assess the level of stress and depression of the patient. It is expected that the next researcher will use another method of data collection, can use the subjective and objective method. And add a longer time to the research process to get more useful results.

**Table:** 

Citation	**Ramanathan, U.; Malaviya, G. N.; Jain, N.; Husain, S. (1991). Psychosocial aspects of deformed leprosy patients undergoing surgical correction. Leprosy Review. 62, 402–409.**	
Study purpose: Was the purpose stated clearly? ☒Yes ☐No	*Outline the purpose of the study*. p.403, Introduction: This prompted us to undertake this preliminary study in order to find out the psychosocial status of the patients with deformities before and after surgical correction and also to assess: (1) The sociodemographic characteristics of leprosy cases who opted for surgical correction for the deformities. (2) The intelligence and personality make-up of these patients (3) The depression and anxiety levels of these patients before and after surgical correction. (4) The expectations from surgery and its fulfilment after operation. *How does the study apply to your research question*? The study assesses how surgical correction of leprosy symptoms can affect the mental wellbeing, specifically depression and anxiety levels, of patients affected by leprosy.	
Literature Was relevant background literature reviewed? ☒Yes ☐No	*Describe the justification of the need for this study*: p.402, Introduction: It has been estimated that approximately 25% of leprosy patients are deformed. Patients with leprosy are very self-conscious of any deformity they have which they feel may advertise to others that they have or have had leprosy. One study concluded that reconstructive surgery helps leprosy patients, at least to some extent, to regain their cosmetic, functional, occupational and economic status in society.	
Design: ☐Randomized (RCT) ☐Cohort ☐Single case design ☒before and after ☐case-control ☐cross-sectional ☐case study	*Describe the study design*. *Was the design appropriate for the study question*? *(e*.*g*., *for knowledge level about this issue*, *outcomes*, *ethical issues*, *etc*.): p.403, Materials and methods: The cases were selected at random from the group opting for corrective surgery and a final sample of 25 patients has been included in the study. The cases were hospitalized for 1 or 2 days as many of them had travelled long distances. This was done to eliminate the effects of travel and exertion. The patients were assessed a week prior to surgery and then every 3 months for 3 successive visits. For psychological evaluation the researchers used Beck’s depression inventory; Taylor’s manifest anxiety scale test; Thematic apperception test (TAT); and Wechsler adult intelligence scale test as modified and adapted to Indian situations by Ramlingaswamy. *Specify any biases that may have been operating and the direction of their influence on the results*: p.408, Discussion: The researchers discuss the importance of pre-counselling prior the surgery, however, no limitations or biases have been reported otherwise.	
Sample N = 25 Was the sample described in detail? ☒Yes ☐No Was sample size justified? ☐Yes ☒No ☐N/A	*Sampling (who; characteristics; how many; how was sampling done*?*) If more than one group*, *was there similarity between the groups*?: p.403, Materials and Methods: The cases were selected at random from the group opting for corrective surgery and a final sample of 25 patients has been included in the study. The cases were hospitalized for 1 or 2 days as many of them had travelled long distances. This was done to eliminate the effects of travel and exertion. The patients were assessed a week prior to surgery and then every 3 months for 3 successive visits. *Describe ethics procedures*. *Was informed consent obtained*?: No information provided.	
Outcomes Were the outcome measures reliable? ☒Yes ☐No ☐Not addressed Were the outcome measures valid? ☒Yes ☐No ☐Not addressed	*Specify the frequency of outcome measurement (i*.*e*., *pre*, *post*, *follow-up)*:	
	*Outcome areas*: Depression level Anxiety level Intelligence level Problem-solving skills SES characteristics	*List measures used*: Beck ’s depression inventory Taylor ’s Manifest Anxiety Scale Wechsler Adult Intelligence Scales (W A IS) Thematic Apperception Test (TA T) Initial assessment after inclusion in the study
Intervention Intervention was described in detail? ☒Yes ☐No ☐Not addressed Contamination was avoided? ☐Yes ☐No ☒Not addressed ☐N/A Cointervention was avoided? ☐Yes ☐No ☒Not addressed ☐N/A	*Provide a short description of the intervention (focus*, *who delivered it*, *how often*, *setting)*. *Could the intervention be replicated in practice*? The included patients were hospitalized for one to two days prior the intervention and were asked to tell their expectations regarding the surgery. Applying the mentioned assessment scales the patients were assessed a week prior the surgery and then every three months for three successive visits. Thereby, the researchers applied the following scales/ assessment tools: Beck’s depression inventory; Taylor’s manifest anxiety scale test; Thematic apperception test (TAT); and Wechsler adult intelligence scale test as modified and adapted to Indian situations by Ramlingaswamy.	
Results Results were reported in terms of statistical significance? ☐Yes ☒No ☐N/A ☐Not addressed Were the analysis method(s) appropriate? ☐Yes ☒No ☐Not addressed Clinical importance was reported? ☒Yes ☐No ☐Not addressed	*What were the results*? *Were they statistically significant (i*.*e*., *p < 0*.*05)*? *If not statistically significant*, *was study big enough to show an important difference if it should occur*? *If there were multiple outcomes*, *was that taken into account for the statistical analysis*? The results were provided in percentage and the difference of cases of depression/anxiety before and after the intervention. However, no statistical significance level or p-value was provided.	
Drop-outs were reported? ☐Yes ☒No	*Did any participants drop out from the study*? *Why*? *(Were reasons given and were drop-outs handled appropriately*?) No information about dropouts was provided.	
Conclusion and Implications Conclusions were appropriate given study methods and results ☒Yes ☐No	*What did the study conclude*? *What are the implications of these results for practice*? *What were the main limitations or biases in the study*? p.408, Discussion: It is suggested that before corrective surgery is undertaken, a proper discussion should take place with the patients about their problems, and the benefits valid limitations of surgery. This will help to improve patient cooperation and motivation and also reduce the frustrations of either side. The postoperative goals should be more realistic for the patients and this should be explained to them like any other cosmetic surgical procedure. It therefore seems essential to include surgical reconstruction in a programme of rehabilitation designed to assist these patients to return to society as useful members. The surgical correction of deformities in leprosy is most rewarding and indispensable for the rehabilitation of the patients who have been deformed due to the disease.	

**Citation**	*Provide the full citation for this article in APA format*: Ramasamy, S., Panneerselvam, S., Govindharaj, P., Kumar, A., & Nayak, R. (2018). Progressive muscle relaxation technique on anxiety and depression among persons affected by leprosy. Journal of Exercise Rehabilitation, 14(3), 375–381. https://doi.org/10.12965/jer.1836158.079	
**Study purpose:** Was the purpose stated clearly? ☒Yes ☐No	*Outline the purpose of the study*. p. 375, Abstract: This study aimed to assess the effectiveness of PMRT in reducing anxiety and depression among the hospitalized leprosy affected person in a tertiary care centre. *How does the study apply to your research question*? The study assesses how progressive muscle relaxation therapy can improve anxiety and depression among persons affected by leprosy.	
**Literature** Was relevant background literature reviewed? ☒Yes ☐No	*Describe the justification of the need for this study*: p.375, Abstract: Anxiety and depression have been found to be increasing among people with leprosy and it may lead to decreased social participation. The progressive muscle relaxation technique (PMRT) is widely used today in choice of treatment for reducing the anxiety and depression. / p.376, Introduction: Relaxation has a significant psychological impact. Through relaxation we can improve specific aspects of our personality, strengthening the positive qualities and changing unwanted habits and attitudes.	
**Design:** ☐Randomized (RCT) ☐Cohort ☐Single case design ☒before and after ☐case-control ☐cross-sectional ☐case study	*Describe the study design*. *Was the design appropriate for the study question*? *(e*.*g*., *for knowledge level about this issue*, *outcomes*, *ethical issues*, *etc*.): p.376, Materials and Methods: A prospective open-label single arm study conducted with 50 leprosy affected people who were admitted for leprosy complications during the period of March to August, 2016 in a tertiary leprosy referral hospital. *Specify any biases that may have been operating and the direction of their influence on the results*: p.379, Discussion: No control group for comparison of the intervention.	
**Sample** N = 50 Was the sample described in detail? ☒Yes ☐No **Was sample size justified?** ☒Yes ☐No ☐N/A	*Sampling (who; characteristics; how many; how was sampling done*?*) If more than one group*, *was there similarity between the groups*?: p.376, Materials and methods: The study includes those aged between 18–60 years, who were willing to participate in the study and able to understand relaxation techniques. Those patients who were practicing other relaxation technique such as yoga and/or on meditation for anxiety or depression were excluded from the study. A total of 50 subjects who are aged between from 18 to 60 years were undergone PMRT during the study period, out of all participants 36 (72%) were males. *Describe ethics procedures*. *Was informed consent obtained*?: p.376, Procedure: Informed consent was taken prior to inclusion in the study from each individual participant. All the collected information about patient was kept confidential.	
**Outcomes** Were the outcome measures reliable? ☒Yes ☐No ☐Not addressed Were the outcome measures valid? ☒Yes ☐No ☐Not addressed	*Specify the frequency of outcome measurement (i*.*e*., *pre*, *post*, *follow-up)*: p.377, Procedure: The anxiety-depression scale assessment was first administered at the time of admission and repeated after 2 weeks. Patients were instructed to continue the same technique and follow-up assessment were done in 6 weeks after the initial intervention.	
	*Outcome areas*: Depression and anxiety level SES characteristics	*List measures used*: Anxiety-depression scale Beck anxiety inventory Semi structured questionnaire
**Intervention** Intervention was described in detail? ☒Yes ☐No ☐Not addressed Contamination was avoided? ☐Yes ☐No ☒Not addressed ☐N/A Cointervention was avoided? ☒Yes ☐No ☐Not addressed ☐N/A	*Provide a short description of the intervention (focus*, *who delivered it*, *how often*, *setting)*. *Could the intervention be replicated in practice*? p.376, Intervention of PMRT: Jacobsen progressive muscle relaxation technique was demonstrated and taught after ensuring that the participants understood about the technique. In first and third steps of the relaxation sequence, the patients were advised to begin the technique from right side of the body followed by left side. In a day, two sessions were conducted in the morning and evening approximately for 30 min. The anxiety-depression scale assessment was first administered at the time of admission and repeated after 2 weeks. Patients were instructed to continue the same technique and follow-up assessment were done in 6 weeks after the initial intervention. p.376, Inclusion and exclusion criteria: Those patients who were practicing other relaxation technique such as yoga and/or on meditation for anxiety or depression were excluded from the study. Those who are alcohol dependence, tobacco chewers and/or smokers were also excluded as they may directly affect psychological status of an individual.	
**Results** Results were reported in terms of statistical significance? ☒Yes ☐No ☐N/A ☐Not addressed Were the analysis method(s) appropriate? ☒Yes ☐No ☐Not addressed Clinical importance was reported? ☒Yes ☐No ☐Not addressed	*What were the results*? *Were they statistically significant (i*.*e*., *p < 0*.*05)*? *If not statistically significant*, *was study big enough to show an important difference if it should occur*? *If there were multiple outcomes*, *was that taken into account for the statistical analysis*?p.378, Results: A statistically significant difference was seen on anxiety beforeand after application of PMR techniques. The anxiety means scoreshowed steady decline from 6.76 at pretest to 3.0 (t = 25.068, P≤ 0.01) at post-test and 1.12 (t = 22.679, P≤0.001) at final follow-up. A statistically significant difference was seen on depression before and after application of PMR technique. The depression means score showed steady decline from 6.92 at pretest to 3.28 (t = 16.082, P≤0.001) at posttest and to 1.16 (t = 18.918, P≤0.001) at final follow-up.	
Drop-outs were reported? ☐Yes ☒No	*Did any participants drop out from the study*? *Why*? *(Were reasons given and were drop-outs handled appropriately*?) No information provided.	
**Conclusion and Implications** Conclusions were appropriate given study methods and results ☒Yes ☐No	*What did the study conclude*? *What are the implications of these results for practice*? *What were the main limitations or biases in the study*? p.379, Discussion: In summary, the PMRT is effective relaxation therapy to reduce the anxiety and depression among hospitalized person with leprosy. This relaxation therapy is easy to learn and practice, does not require trained professional or any equipment to facilitate the training. This study proved as a valid treatment option for hospitalized person with leprosy in minimizing the anxiety and depression related symptoms and to benefit the psychosocial well-being of leprosy affected patients.

**Table:** 

**Citation**	*Provide the full citation for this article in APA format*: Geroge, A., Khora, T., Das, P., & Rao, P. S. (2013). Nursing interventions to manage anxiety levels of female inpatients admitted first time in a leprosy hospital. Indian journal of leprosy, 85(1), 19–25.	
**Study purpose:** Was the purpose stated clearly? ☒Yes ☐No	*Outline the purpose of the study*. p.19, Introduction: the study investigates the role of nurses in managing various anxieties of female patients admitted for the first time in a leprosy referral hospital. *How does the study apply to your research question*? The study assesses how a nursing intervention helps to improve the anxiety levels of female leprosy patients.	
**Literature** Was relevant background literature reviewed? ☒Yes ☐No	*Describe the justification of the need for this study*: p. 19, Introduction: Hospital admissions produce high levels of anxieties or most patients but especially among women in India. These anxieties are exacerbated when the women are suffering from a stigmatised disease such as leprosy. Nurses play a key role in managing such situations successfully. Such studies are useful and often essential in Indian setting but rarely reported.	
**Design:** ☐Randomized (RCT) ☐Cohort ☐Single case design ☒before and after ☐case-control ☐cross-sectional ☐case study	*Describe the study design*. *Was the design appropriate for the study question*? *(e*.*g*., *for knowledge level about this issue*, *outcomes*, *ethical issues*, *etc*.): p. 20, Materials and Methods: The nursing care plan consisted of assessment, nursing diagnosis, interventions, implementations and evaluations. By applying the Hamilton Anxiety Rating Scale the anxiety levels of the women affected by leprosy were measured before the nursing intervention and after. The nursing interventions were planned and formulated according to the assessment of each individual and according to the care each person demanded. *Specify any biases that may have been operating and the direction of their influence on the results*: p.24, Discussion: The researchers report that there could have been more variability in the interventions.	
**Sample** N = 40 Was the sample described in detail? ☒Yes ☐No **Was sample size justified?** ☒Yes ☐No ☐N/A	*Sampling (who; characteristics; how many; how was sampling done*?*) If more than one group*, *was there similarity between the groups*?: p.20, Materials and Methods: All female leprosy patients in the age group of 20 to 60 years admitted to the hospital for the first time were included. However, physically and mentally challenged and revisits were excluded. Based on mathematical calculations the minimum sample size for the study was determined as 40 female leprosy patients. *Describe ethics procedures*. *Was informed consent obtained*?: No ethical procedures were explained.	
**Outcomes** Were the outcome measures reliable? ☒Yes ☐No ☐Not addressed Were the outcome measures valid? ☒Yes ☐No ☐Not addressed	*Specify the frequency of outcome measurement (i*.*e*., *pre*, *post*, *follow-up)*:Pre- and Post anxiety levels	
	*Outcome areas*: Anxiety levels SES characteristics	*List measures used*: Hamilton assessment scale Patient registers
**Intervention** Intervention was described in detail? ☒Yes ☐No ☐Not addressed Contamination was avoided? ☐Yes ☐No ☒Not addressed ☐N/A Cointervention was avoided? ☐Yes ☐No ☒Not addressed ☐N/A	*Provide a short description of the intervention (focus*, *who delivered it*, *how often*, *setting)*. *Could the intervention be replicated in practice*? The nursing care plan consists of assessment, nursing diagnosis, intervention, implementation and evaluation. The specific steps are explained in the study. The nursing interventions were planned and formulated according to the assessment of each individual and according to the care each person demanded. Patients who had anxiety regarding their family or their body image were kept occupied with some woks like bandage making or assisting disabled patients etc.	
**Results** Results were reported in terms of statistical significance? ☒Yes ☐No ☐N/A ☐Not addressed Were the analysis method(s) appropriate? ☒Yes ☐No ☐Not addressed Clinical importance was reported? ☒Yes ☐No ☐Not addressed	*What were the results*? *Were they statistically significant (i*.*e*., *p < 0*.*05)*? *If not statistically significant*, *was study big enough to show an important difference if it should occur*? *If there were multiple outcomes*, *was that taken into account for the statistical analysis*? p.23, Findings: The findings were statistically highly significant and emphasises the importance of well-planned individualistic nursing interventions to reduce or minimize the anxiety level of female leprosy patients admitted the first time in the hospital. The assessment done post-intervention indicated that only 26 patients had mild anxiety, 12 still had moderate anxiety and only 2 continued to have severe anxiety.	
Drop-outs were reported? ☐Yes ☒No	*Did any participants drop out from the study*? *Why*? *(Were reasons given and were drop-outs handled appropriately*?) No information provided.	
**Conclusion and Implications** Conclusions were appropriate given study methods and results ☒Yes ☐No	*What did the study conclude*? *What are the implications of these results for practice*? *What were the main limitations or biases in the study*? p.24, Discussion: The study suggest that well planned nursing interventions can have far reaching benefits to the patients and accelerate therapeutic outcomes. Relieving anxieties and ensuring the patients’ coping skills through nursing interventions are critical in chronic disease hospitalization, especially for women patients.	

#### 4) NICE checklist–qualitative studies

**Table:** 

**Study identification:**	**Authors**: Floyd-Richard, M.; Gurung, S. **Title**: Stigma reduction through group counselling of persons affected by leprosy–a pilot study **Reference and year of publication:** Leprosy Review, 2000	
**Checklist completed by:**	Ann-Kristin Bonkass	
**Theoretical approach**		
**1. Is a qualitative approach appropriate?** For example: • Does the research question seek to understand processes or structures, or illuminate subjective experiences or meanings? • Could a quantitative approach better have addressed the research question?	Appropriate ☒ Inappropriate☐ Not sure ☐	**Comments:** p. 500; Introduction: “Psychosocial assessments for all inpatients were provided and then individual counselling offered to those needing specific help in emotional, social, spiritual or reproductive health.”
**2. Is the study clear in what it seeks to do?** For example: • Is the purpose of the study discussed aims/objectives/research question/s? • Is there adequate/appropriate reference to the literature? • Are underpinning values/assumptions/theory discussed?	Clear ☒ Unclear☐ Mixed☐	**Comments**: p. 500; Introduction: “Due to the recurring themes of stigmatized patients we decided to conduct a pilot study on the efficacy of group counselling in dealing with stigmatization issues”./ Materials and Methods: “If they demonstrate one or more of the following they are offered group therapy: their own recognition that their low self-esteem is due to stigmatization, they have been rejected by their families, or despondent but not clinically depressed.”
**Study design**		
**3. How defensible/rigorous is the research design/methodology?** For example: • Is the design appropriate to the research question? • Is a rationale given for using a qualitative approach? • Are there clear accounts of the rationale/justification for the sampling, data collection and data analysis techniques used? • Is the selection of cases/sampling strategy theoretically justified?	Defensible☒ Indefensible☐ Not sure☐	**Comments**: p. 500, Materials and Methods: “Groups composed of five to seven individuals meet for 2-h sessions each week for 5 weeks. In these groups people are encouraged to remember things from three phases in their lives: before contracting leprosy, after contracting leprosy, and life as an inpatient at GPH.” p. 500, Introduction: “We surmised that group counselling would help people to: develop relationships as they described their similar sufferings, educate them on the common effects of stigmatisation (ie. social isolation, depression and decreased self-esteem), and encourage them to think constructively on how they can begin to rebuild their self-esteem and thus, their lives. “
**Data collection**		
**4. How well was the data collection carried out?** For example: • Are the data collection methods clearly described? • Were the appropriate data collected to address the research question? • Was the data collection and record keeping systematic?	Appropriately☐ Inappropriately☐ Not sure/inadequately reported☒	**Comments**: p. 500, Materials and Methods: “Groups composed of five to seven individuals meet for 2-h sessions each week for 5 weeks. There were 22 counselling groups from March 1 994 to February 1 998. Seventy-four percent (86/1 17) had WHO impairment scores of 1 or 2.”
**Trustworthiness**		
**5. Is the role of the researcher clearly described?** For example: • Has the relationship between the researcher and the participants been adequately considered? • Does the paper describe how the research was explained and presented to the participants?	Clearly described ☒ Unclear☐ Not described☐	**Comments**: p. 500, Introduction/ Materials and Methods: “In October 1 993, the Counselling Department at Green Pastures Hospital (GPH), Pokhara, Nepal was started to help heal and rebuild people psychologically and socially. Psychosocial assessments for all inpatients were provided and then individual counselling offered to those needing specific help in emotional, social, spiritual or reproductive health.” “Within the first week after admission patients are given a psychosocial assessment. If they demonstrate one or more of the following they are offered group therapy […]”
**6. Is the context clearly described?** For example: • Are the characteristics of the participants and settings clearly defined? • Were observations made in a sufficient variety of circumstances? • Was context bias considered?	Clear☐ Unclear☒ Not sure☐	**Comments**: p. 500, Materials & Methods: “A database was started including simple leprosy demographics and specific questions regarding WHO Impairment Scores, marital status, and family support.”
**7. Were the methods reliable?** For example: • Was data collected by more than 1 method? • Is there justification for triangulation, or for not triangulating? • Do the methods investigate what they claim to?	Reliable☐ Unreliable☐ Not sure☒	**Comments**: p. 500, Introduction: “Due to time restraints and the recurring themes of stigmatized patients we decided to conduct a pilot study on the efficacy of group counselling in dealing with stigmatization issues.”
**Analysis:**		
**8. Is the data analysis sufficiently rigorous?** For example: • Is the procedure explicit–i.e. is it clear how the data was analysed to arrive at the results? • How systematic is the analysis, is the procedure reliable/dependable? • Is it clear how the themes and concepts were derived from the data?	Rigorous☐ Not rigorous☐ Not sure/not reported☒	**Comments**: No comments provided.
**9. Is the data ’rich’?** For example: • How well are the contexts of the data described? • Has the diversity of perspective and content been explored? • How well has the detail and depth been demonstrated? • Are responses compared and contrasted across groups/sites?	Rich☒ Poor☐ Not sure/not reported☐	**Comments**: p.500, Materials and Methods: Group therapy within the 5-week cycle was done by a pattern described in the results for each week (Week 1–5).
**10. Is the analysis reliable?** For example: •Did more than 1 researcher theme and code transcripts/data? • If so, how were differences resolved? • Did participants feed back on the transcripts/data if possible and relevant? • Were negative/discrepant results addressed or ignored?	Reliable☐ Unreliable☐ Not sure/not reported☒	**Comments**: No comments provided.
**11. Are the findings convincing?** For example: • Are the findings clearly presented? • Are the findings internally coherent? • Are extracts from the original data included? • Are the data appropriately referenced? • Is the reporting clear and coherent?	Convincing☒ Not convincing☐ Not sure☐	**Comments**: Data is being convincingly and adequately presented in the paragraphs of the different counselling weeks. The researchers provide a close description of the feelings and emotions of the participants during the sessions (Floyd-Richard et al. 500ff.).
**12. Are the findings relevant to the aims of the study?**	Relevant ☒ Irrelevant ☐ Partially relevant ☐	**Comments**: p.503, Discussion: “From our pilot study we conclude that group counselling is a time efficient and productive method of counselling to use in reducing the effects of stigmatization on people with leprosy.”
**13. Conclusions** **Is there adequate discussion of any limitations encountered?** For example: •How clear are the links between data, interpretation and conclusions? • Are the conclusions plausible and coherent? • Have alternative explanations been explored and discounted? • Does this enhance understanding of the research topic? • Are the implications of the research clearly defined?	Adequate☐ Inadequate☒ Not sure☐	**Comments**: p. 503, Discussion: “Although we believe that group counselling is an efficient and productive method to use for this group of people, our outcome measures are weak.” No further limitations or interpretations were provided.
**Ethics**		
**14. How clear and coherent is the reporting of ethics?** For example: • Have ethical issues been taken into consideration? • Are they adequately discussed e.g. do they address consent and anonymity? • Have the consequences of the research been considered i.e. raising expectations, changing behaviour? • Was the study approved by an ethics committee?	Appropriate☐ Inappropriate☐ Not sure/not reported☒	**Comments**: No comments provided.
**Overall assessment**		
**As far as can be ascertained from the paper, how well was the study conducted? (see guidance notes)**	+	**Comments**: Most of the categories have been checked as fulfilled. However, there are a number of questions which can not be answered as there are no information provided in the study. Hence, the quality is can be rated as medium/low.
**Study identification:**	**Authors**: Susanto, T.; Dewi, E.; Rahmawati, I. **Title**: The experiences of people affected by leprosy who participated in self-care groups in the community: A qualitative study in Indonesia **Reference and year of publication:** Leprosy Review, 2017	
**Checklist completed by:**	Ann-Kristin Bonkass	
**Theoretical approach**		
**1. Is a qualitative approach appropriate?** For example: • Does the research question seek to understand processes or structures, or illuminate subjective experiences or meanings? • Could a quantitative approach better have addressed the research question?	Appropriate ☒ Inappropriate☐ Not sure ☐	**Comments:** p. 544; Methods: “This study uses a phenomenological, qualitative approach and descriptive design to explore and understand people’s everyday life experiences. The feelings of people affected by leprosy who participated in SCGs were explored through descriptive phenomenology in order to explain the experiences of each person undergoing care in detail, broadly and profoundly.”
**2. Is the study clear in what it seeks to do?** For example: • Is the purpose of the study discussed aims/objectives/research question/s? • Is there adequate/appropriate reference to the literature? • Are underpinning values/assumptions/theory discussed?	Clear ☒ Unclear☐ Mixed☐	**Comments**: p. 544; Introduction: “[…] it is necessary to conduct qualitative research with descriptive phenomenology designed to identify the meaning of experiences of people affected by leprosy in joining SCGs. Therefore, the aim of this study was to understand the experiences of those participating in SCGs, with the aim improving the functions of SCGs to resolve self-care problems in the community. “
**Study design**		
**3. How defensible/rigorous is the research design/methodology?** For example: • Is the design appropriate to the research question? • Is a rationale given for using a qualitative approach? • Are there clear accounts of the rationale/justification for the sampling, data collection and data analysis techniques used? • Is the selection of cases/sampling strategy theoretically justified?	Defensible☒ Indefensible☐ Not sure☐	**Comments**: p. 544, Methods: “This study uses a phenomenological, qualitative approach and descriptive design to explore and understand people’s everyday life experiences. “
**Data collection**		
**4. How well was the data collection carried out?** For example: • Are the data collection methods clearly described? • Were the appropriate data collected to address the research question? • Was the data collection and record keeping systematic?	Appropriately☒ Inappropriately☐ Not sure/inadequately reported☐	**Comments**: p. 544, Participants: “A purposive sampling strategy was used to recruit participants from two out of 17 SCGs in the community. Formal letters were sent to PHNs of the two SCGs in Jember, East Java, Indonesia, requesting them to inform those affected by leprosy under their care about focus group interviews, which the investigators had arranged. Data was collected using a semi-structured interview guide with open-ended questions.” Further explanation of the inclusion criteria.
**Trustworthiness**		
**5. Is the role of the researcher clearly described?** For example: • Has the relationship between the researcher and the participants been adequately considered? • Does the paper describe how the research was explained and presented to the participants?	Clearly described ☒ Unclear☐ Not described☐	**Comments**: p. 545, Data Collection: “The purpose and procedure of the study were explained to the participants and questions were invited. In this study, researchers used therapeutic communication techniques to obtain information from participants.”
**6. Is the context clearly described?** For example: • Are the characteristics of the participants and settings clearly defined? • Were observations made in a sufficient variety of circumstances? • Was context bias considered?	Clear☒ Unclear☐ Not sure☐	**Comments**: p. 544f., Participants: The sociodemographic characteristics of the study sample is described in depth with mean age, gender distribution and participant’s education level.
**7. Were the methods reliable?** For example: • Was data collected by more than 1 method? • Is there justification for triangulation, or for not triangulating? • Do the methods investigate what they claim to?	Reliable☒ Unreliable☐ Not sure☐	**Comments**: p. 545, Data analysis: “The investigators (TS, EID and IR) collected and guided the focus group interviews and then transcribed the discussions verbatim. / Discussion: Self-help groups are very helpful for people affected by leprosy in following short-term and long-term therapeutic regimens of leprosy treatment in the community.”
**Analysis:**		
**8. Is the data analysis sufficiently rigorous?** For example: • Is the procedure explicit–i.e. is it clear how the data was analysed to arrive at the results? • How systematic is the analysis, is the procedure reliable/dependable? • Is it clear how the themes and concepts were derived from the data?	Rigorous☒ Not rigorous☐ Not sure/not reported☐	**Comments**: p. 545, Data analysis: “The investigators (TS, EID and IR) collected and guided the focus group interviews and then transcribed the discussions verbatim. The data were analysed through seven processes” (explained afterwards)./ Results; p.546: “The analysis identified five themes surrounding people’s experience of participating in SCGs in the community: self-perceived condition, adherence to treatment, ability to do personal care, the kind of help and services received, and acceptance and support of the person affected by leprosy.”
**9. Is the data ’rich’?** For example: • How well are the contexts of the data described? • Has the diversity of perspective and content been explored? • How well has the detail and depth been demonstrated? • Are responses compared and contrasted across groups/sites?	Rich☒ Poor☐ Not sure/not reported☐	**Comments**: p.546, Results: “The analysis identified five themes surrounding people’s experience of participating in SCGs in the community: self-perceived condition, adherence to treatment, ability to do personal care, the kind of help and services received, and acceptance and support of the person affected by leprosy. “/ Discussion; p. 550: “Our study highlighted the ability of a person affected by leprosy to do personal care, with a focus on personal care of skin and wounds, and prevention of disability. “
**10. Is the analysis reliable?** For example: •Did more than 1 researcher theme and code transcripts/data? • If so, how were differences resolved? • Did participants feed back on the transcripts/data if possible and relevant? • Were negative/discrepant results addressed or ignored?	Reliable☒ Unreliable☐ Not sure/not reported☐	**Comments**: p. 544; Methods: The participants were informed that they could withdraw from the study at any time. Participants were divided into two groups for interview.; Data analysis: The investigators (TS, EID and IR) collected and guided the focus group interviews and then transcribed the discussions verbatim.
**11. Are the findings convincing?** For example: •Are the findings clearly presented? • Are the findings internally coherent? • Are extracts from the original data included? • Are the data appropriately referenced? • Is the reporting clear and coherent?	Convincing☒ Not convincing☐ Not sure☐	**Comments**: Results, p. 546ff.: Personal quotes of participants are provided, divided in the before explained sub-categories. Clear reporting of the topics discussed in the self-help groups.
**12. Are the findings relevant to the aims of the study?**	Relevant ☒ Irrelevant ☐ Partially relevant ☐	**Comments**: p.503, Discussion: “From our pilot study we conclude that group counselling is a time efficient and productive method of counselling to use in reducing the effects of stigmatization on people with leprosy.”
**13. Conclusions** **Is there adequate discussion of any limitations encountered?** For example: •How clear are the links between data, interpretation and conclusions? • Are the conclusions plausible and coherent? • Have alternative explanations been explored and discounted? • Does this enhance understanding of the research topic? • Are the implications of the research clearly defined?	Adequate☒ Inadequate☐ Not sure☐	**Comments**: p. 551, Conclusion: “This study explores the experiences of people affected by leprosy, while attending SCGs in the community, which aim to help meet their basic needs and encourage self-care, including physical, psychological, social, economic, cultural, and spiritual aspects.”/ p. 551, Implications for community and public health nursing: “The SCGs could be improved in functioning for those affected by leprosy in their community to meet their basic needs and self-care training, including physical, psychological, social, economic, cultural, and spiritual aspects. “
**Ethics**		
**14. How clear and coherent is the reporting of ethics?** For example: • Have ethical issues been taken into consideration? • Are they adequately discussed e.g. do they address consent and anonymity? • Have the consequences of the research been considered i.e. raising expectations, changing behaviour? • Was the study approved by an ethics committee?	Appropriate☒ Inappropriate☐ Not sure/not reported☐	**Comments**: p. 545, Data collection: “This study was approved by the Research Department of the University of Jember. The participants were informed that they could withdraw from the study at any time. Participants were divided into two groups for interview.”
**Overall assessment**		
**As far as can be ascertained from the paper, how well was the study conducted? (see guidance notes)**	++	**Comments**: All of the categories have been fulfilled by the study. Hence, one can conclude that the overall quality of the study is high.

## 3 Results

Due to the limited availability of psychosocial interventions to promote the mental wellbeing of PALs merely 17 studies were identified for this systematic review. The chosen studies included data from different countries, namely: Brazil (n = 1), India (n = 7), Nepal (n = 3), Indonesia (n = 4), Egypt (n = 1) and Taiwan (n = 1). As there is a variety of psychosocial interventions available, categories to achieve a better overview of the intervention types were created. The interventions were divided into four categories: educational interventions, counselling strategy, cognitive behavioural therapy (CBT) and other or technology supported interventions. By creating categories, the author ensured that the findings of the included studies are appropriately presented. The categorization facilitates the comparability of the findings, in order to make an educated statement about what intervention would be useful in the Indian setting.

### 3.1. Psychoeducational interventions

Knowledge is an important factor when it comes to stigmatisation of PALs. Multiple papers have reported how a lack of knowledge can affect the own perception of a disease, such as leprosy [18]. The increase in knowledge around the transmission and symptoms of leprosy would facilitate early detection, which is essential to reduce social and physical consequences of the illness [18]. The literature selection identified two papers which describe effective psychoeducational interventions, namely Mardhiyah [12] and Ahmed & Mohamed [19]. The study conducted by Mardhiyah [12] assessed the knowledge levels of their participants before and after the educational intervention. Based on the data conducted pre and post intervention, the psychoeducational intervention was proven effective. However, it was more effective in participant two and three than in participant one, as their SRQ score was still at six, while the other two had a significantly lower score (4 and 3 respectively). Hence, the set objective for the SRQ score was achieved for two out of three participants. Furthermore, participants self-reported a decrease in psychological distress after the intervention, and increased knowledge [12].

The study by Ahmed & Mohamed [19] was conducted in a Dermatology Hospital in Egypt. To assess the effectiveness of the intervention, pre and post measurements were taken from the individuals, also evaluating the patient’s self-care practices [19]. Thereby, the used measures were validated by a pilot study, performed beforehand. While the majority of the participants (66.7%) had a poor knowledge level regarding leprosy prior the intervention, their levels significantly increased after the educational intervention that was performed by the nurses of the hospital. Thus, 27.8% of the participants achieved an average knowledge level and 61.1% even a good level [19]. Self-care practices improved as well after the educational intervention. Additionally, the psychological problems significantly reduced after the intervention. A statistical difference was further seen in the reduced stigmatisation participants encountered daily. Not only were their psychological problems reduced, but also their psychological needs were more fulfilled after the intervention, as the participants acquired a better understanding of the disease itself and how it affects the body [19].

Overall, the educational interventions evaluated by Mardhiyah [12] and Ahmed & Mohamed [19] were proven effective at the scale they were applied. When developing psychoeducation strategies, like the ones described, it is important to keep in mind the vulnerable situation participants are in.

### 3.2 Counselling interventions

As there are a number of different intervention types available for the mental wellbeing of leprosy patients, counselling interventions are rather popular among researchers. This type of intervention has been proven effective in other health issues, especially for psychological conditions such as anxiety and depressive disorders [20]. As indicated by some of the included studies, counsellors can also be lay people or volunteer, which makes this kind of strategy not only cost-effective but also more trustworthy for affected individuals [21].

This systematic review included seven studies that used counselling interventions to improve the mental wellbeing of leprosy patients and to reduce stigma-related harm. Bhat et al. [20] assessed how counselling strategies improved the social participation of PALs in 120 patients in Kashmir and Jammu, India. The aim of the counselling intervention was to provide accurate information about the disease itself and how it can be managed. A substantial reduction was observed in the participation restriction scores, which demonstrates to what extent people feel excluded from social activities. Therefore, the percentage of participants feeling extreme restriction decreased from 22.82% to 8.33% while a feeling of no restriction increased from 0% to 3.33%. While the intervention affected people from all demographic backgrounds, the following factors were significant contributors to an increased participation restriction score: being female, aged over 40 years, disabled, unmarried and unemployed [20].

Floyd-Richard & Gurung [22] conducted their research at the Green Pastures Hospital in Pokhara, Nepal. The participants were divided into single sex and children’s groups, together with a counselling psychologist and a nurse. During the sessions, difficult moments of participants’ lives were discussed, as well as resilience strategies to meet their own needs. While the intervention was evaluated as effective in reducing the impact of stigmatisation on leprosy affected persons, the outcome measures were weak. However, by connecting with group members, individuals felt less alone with their problems and fears [22]. To improve the intervention, Floyd-Richard & Gurung [22] recommended the inclusion of recovered leprosy patients as counsellors, in order to build a stronger connection with the participants. Additionally, the researchers admitted that counselling alone may be insufficient to meet the psychological needs of participants, thus, they suggest a combined approach with health educational methods.

While Floyd-Richard & Gurung [22] and Bhat et al. [23] solely used counselling strategies, Dadun et al. [23] added socio-economic development (SED) along with a contact task to the intervention, called “Stigma Assessment and Reduction of Impact’’ Project (SARI) [23]. The controlled trial was performed in Indonesia. While the first session was performed by a professional counsellor, the remaining sessions were provided by lay- or peer-counsellors. The counselling part of the intervention consisted of five sessions on individual, group, and family basis. Lastly, the contact section between the affected people and community members aimed at reducing negative feelings and stereotypes towards leprosy. All three interventions were proven to be effective to improve QoL (p = 0.013), participation restriction (p = 0.001) and stigma (p = 0.001) within the intervention groups. However, only the counselling and the SED intervention was found to significantly improve the QoL of participants (p = 0.036). Social stigma significantly reduced during all three interventions (p = 0.002; p = 0.001 and p = 0.001). Hence, it can be said that the intervention had a beneficial impact on the outcomes within the intervention groups [23].

In Nepal, Jay et al. [21] implemented ten self-help groups for 98 participants throughout different districts where high rates of disability due to leprosy were found. The statistical analysis indicated that there is a positive association between having access to groups and a reduced internalised stigma. The researchers further found a positive association between increased identification with the self-help group and improved psychological wellbeing. Better access to multiple groups was indirectly significantly associated with improved psychological wellbeing (p = 0.001) [21]. As can be seen from the study, having access to social activities within multiple groups, as well as the feeling of belonging to a certain group can help to improve the mental wellbeing of affected leprosy patients.

The rights-based counselling module (RBCM) by Lusli et al. [2] is a much-cited study that, in addition to leprosy patients, further included their family members in the Cirebon District of Indonesia. The counseling was divided into individual, group and family sessions and performed by 23 trained peer and lay counselors. During the RBCM 198 participants received counseling. After the intervention, all of the measures indicated a positive effect of the approach. The SARI scale (p = 0.001) as well as the participation restriction scale (p = .001) decreased while the participants’ QoL (p = .0.001) increased after the counselling sessions. Additionally, when testing for confounders, a significant effect between reduced PSS and SSS score and sex were found, as the counselling intervention was more effective in women [2]. The qualitative data assessment reported similar effects of the intervention, as participants felt more powerful against negative feelings and enacted stigma from others.

Another study conducted in Indonesia was introduced by Susanto et al. [24], aiming at assessing the experiences of PALs after joining self-care groups (SCG). The qualitative data analysis showed that SCG helped to support participant’s ability to take care of themselves and their needs. It further helped affected people to accept themselves and to receive the social acceptance they needed. Hence, the assessed SCG effectively helped PALs to improve their QoL by helping them to become an active member of society again, having an occupation, as well as feeling acceptance from people around them [24].

Van’t Noordende et al. [18] conducted a family-based intervention which was based in two sites, the rural area Odisha and the urban area Telangana in India. The family-based counselling intervention aimed at supporting the participant’s protective abilities and resilience. This format was chosen, as family members and close friends often act as a bedrock of wellbeing and identity of affected persons. The sessions included a problem-solving and action learning approach to emphasize the im-portance of social relationships and family support as well as recognizing spiritual beliefs. All individuals from the Odisha state had a higher WHOQoL BREF (p = 0.0001) which was significantly associated with the intervention. Participants from Telangana also experienced a statistically significant increase in the QoL score (p = 0.004) except the family members of the leprosy patients (p = 0.108). Based on the study’s findings the intervention can be evaluated as effective in increasing QoL and resilience in leprosy patients and their family members. The discrepancy between the different outcomes in the geographical areas can be explained by the varying community demographics and levels of social relationships [18].

The last study was conducted by Jay et al. [25], following an empowerment approach and a group-based initiative. The findings are aligning with results from previous studies and indicate that social belonging to a group has a positive effect on participant’s resilience (p = 0.01), as well as better self- acceptance (p = 0.05). Hence, giving people a sense of belonging through self help groups has a positive effect on people’s psychological well being. As can be seen, all the included studies using a counselling approach came to the same conclusion of it being an effective intervention to reduce stigma and improve the mental wellbeing in PALs. While the studies used similar methods to collect data, the approaches varied slightly as some included family members and others included rights-based counselling strategies.

### 3.3 Cognitive behavioural therapy

Cognitive behaviour therapy has been widely recognized in recent years, as it has been proven effective in a number of mental disorders, such as bipolar disorder, depression and schizophrenia. The goal of the therapy is for patients to regain their confidence and hope in order to reenter social life. Thereby, there are different types of CBT, as will be seen in the results of the included studies, for instance a muscle relaxation technique [26].

The author identified four suitable interventions that address effective CBT. The first study by Rahmawati & Yuniarti [27] assessed the impact of CBT on the level of depression of 25 persons suffering from leprosy. The statistical test showed significant reduction in the participant’s depression level after the intervention. The results were statistically significant (p = 0.001), meaning that CBT can be seen as effective in reducing depression in leprosy patients. Therefore, moderate depression reduced from 20% to 4% and participants with No depression increased from 0% to 12%. However, Rahmawati & Yuniarti [27] mention that the majority of the participants had merely elementary education (52%) which may have been a limitation for a better outcome of the study. Thus, they recommend the complementary use of health educational interventions together with CBT [27].

A slightly different approach to CBT was presented by Ramasamy et al. [26], as they evaluated the effectiveness of a progressive muscle relaxation technique (PMRT) on depression and anxiety levels of 50 leprosy patients. Furthermore, the participants’ depression and anxiety level were measured through a self-developed questionnaire during face-to-face interviews. After explaining the exercise to all participants, they were asked to perform this technique twice a day for the following five to six days under supervision of physiotherapists. After statistical analysis, a significant difference between pre- and post-test scores was seen for both depression and anxiety (both p = 0.001). Additionally, the PMRT was deemed effective among people suffering from psychological disorders, such as depression and anxiety [26].

A quantitative study by Leite & Caldeira [28] conducted in Brazil, appraised the effectiveness of therapeutic workshops on patients QoL and depression level. In order to assess the participant’s psychological wellbeing, the researchers used the WHOQoL BREF as well as the BDI questionnaire at baseline and six months after the intervention. The analysis indicated that there was a significant reduction of depression levels among the participants as well as an increase in the psychological QoL domain of the WHOQoL BREF (p = 0.001). However, the workshops were only effective to reduce the level of moderate depression (p = 0.001) and no depressive symptoms at all (p = 0.001). The researchers justify the inability of the intervention to improve severe depression (p = 0.557) by saying that these patients are unresponsive to workshops like this and the primary treatment option for them should be antidepressant medications. This indicates the limitations of the CBT approach, whereby researchers need to adapt the intervention in order to help everyone [28].

The study by Su et al. [29] was conducted in a Taiwanese leprosy sanatorium, by appraising the effectiveness of reminiscence group therapy in 129 elderly leprosy patients. However, the data regarding the patients’ cognitive functions was not analysed as this did not answer the research questions. The intervention lasted for 24 weeks, whereby the experimental group attended three group sessions of the reminiscence therapy each week and the control group merely had individual interviews. Su et al. define reminiscence therapy as a non‐ pharmacological intervention which “focuses the therapeutic theme on previously experienced events and systemically reviews subjects’ lifestyle and life stages” [29]. At baseline, 27 of the participants were suspected of suffering from depression based on the GDS-SF score. However, after the intervention, the analysis indicated a statistically significant reduction of depression level among the elderly leprosy patients in the intervention group (p = 0.02). There was no statistically significant reduction in the depression level in participants of the control group. Nevertheless, the reminiscence group therapy was effective in reducing the depression level of PALs in the Taiwanese leprosy hospital [29].

### 3.4 Other interventions/ technology supported interventions

While the most established intervention types have been mentioned so far, there are some approaches which were unable to match with one of the categories above. Hence, the author summarised the three remaining studies here [30–32]. As seen in the previous chapters, educational, CBT or counselling interventions do not always reach every participant. Hence, it is important to adapt interventions and to combine different approaches, as can be seen in the following three studies.

This was demonstrated with the study conducted by Ramanathan et al. [31] that combines a counselling strategy with surgical correction of the physical symptoms of leprosy patients to reduce anxiety and depression levels. The study was performed in the Central Institute for Leprosy in Agra, India and included a study population of 25 participants. Additionally, to the surgical intervention, researchers provided psychiatric assistance to boost self-confidence and awareness. To assess the patients’ anxiety and depression level, the BDI, “Taylor’s Manifest Anxiety Scale” [31], as well as an Intelligence Scale were applied. The anxiety and depression levels reduced significantly between pre and post intervention. Severe anxiety symptoms reduced from 11 to 5 while severe depression symptoms reduced from 9 to 3. Patients with no anxiety increased from 5 to 15 and patients with no depression symptoms from 7 to 15. After the study’s completion Ramanathan et al. [31] suggested a better inclusion of educational and informational approaches to achieve a better outcome of a decreased psychological burden.

Dossa et al. [30] conducted another study in India, making use of a technique widely used in neurology, the so-called mental imagery [30]. The researcher included 34 participants which were first assessed with the Depression, Anxiety and Stress Scale-21. While the control group received a conventional aerobic exercise programme, the intervention group received the same exercise programme paired with mental imagery sessions. After the intervention, statistical analysis revealed a significant reduction in depression (p = 0.0001), anxiety (p = 0.0002) and stress levels (p = 0.0001) of the intervention group. However, after comparing the results between the control and intervention group only depression levels reduced significantly (p = 0.0037). This indicates that mental imagery is merely effective in reducing depression, but not anxiety or stress [30].

While in previous studies mostly the respective researchers administered the intervention, Geroge et al. [32] focused on an important professional group in terms of healthcare provision—nurses. The study focused on improved nursing intervention to reduce psychological symptoms in 40 female leprosy patients who were admitted to a leprosy referral hospital in India. The nursing intervention entailed individual assessments of the participants followed by 20–30 minutes sessions. Thereby, the nurses tried to address fears and insecurities of the women by applying a relaxation therapy to patients that felt anxious. Geroge et al. [32] found a statistically significant reduction in the anxiety score of the female participants of all age groups (p = 0.01). Anxiety levels decreased most significantly in older women, aged over 40 (p = 0.0001). This study demonstrates the importance of nurses throughout the healthcare provision of leprosy patients, not only for their physical symptoms but also to facilitate the patient’s mental wellbeing [32].

## 4 Discussion

The 17 included studies demonstrate globally available interventions and their effectiveness to improve the mental wellbeing and QoL of PALs. The research reveals a diverse array of effective approaches, with some suggesting that combining multiple interventions may yield better results [30,25,23,31]. It has been calculated by the WHO, that the indirect and direct economic costs produced by mental disorders are expected to surpass US$ 6 trillion by the year 2030, globally. Thus, providing effective mental health interventions is highly cost-effective as it prevents the deterioration or even emergence of psychological symptoms and hence, makes cost-intensive treatment unnecessary [33,18].

As already mentioned, to find an appropriate intervention, possible conflations of approaches need to be considered. A study by Barakat & Zaki [34] suggests a combined intervention with a counselling and psychoeducational approach. Thereby, researchers need to keep in mind the different levels interventions can be applied on: the intrapersonal level, aiming at individual characteristic; the interpersonal level, that targets relationships between patients and family members or friends; the community level, whereby a certain population group is being addressed and lastly the institutional level, which focuses on laws and regulations that are in place [23]. It is important that the educational interventions aim at community level, while others should be merely focused on the individual. Thus, counselling as well as CBT strategies should be applied on an intrapersonal or possibly interpersonal level, as these interventions are only suitable for a smaller scope [2].

In order to make counselling interventions more cost-effective, the inclusion of lay and peer counsellors or the creation of SCGs, as done by Lusli et al. [2] or Susanto et al. [24], could play a crucial role when it comes to the provision of counselling interventions. Thereby, it could be useful to train people who have recovered from leprosy to provide counselling as this might increase the effectiveness of the intervention due to a higher trustworthiness of the counsellors towards the participants [2]. Furthermore, the introduced interventions by Ramanathan et al. [31] and Dossa et al. [30] show that innovative approaches, like mental imagery or surgical corrections, can be used to promote mental wellbeing in leprosy patients. However, the authors of both studies recommend a complementary intervention with an educational and/ or counselling method. This emphasises the need for more focused research towards the inclusion of more innovative techniques that can be combined with proven approaches to achieve better outcomes. This can be understood as a call for novel innovations and the application of more technology-based interventions as digitalisation facilitates the development of useful apps and websites [35,36].

Several of the included studies found confounding associations between the outcome of their respective intervention and gender [19,20,29]. These findings are supported by a study by Tare et al. (2021) which indicated that women affected by leprosy have a lower QoL due to their diagnosis than men. Additionally, older age has been mentioned to be a confounder in the effectiveness of the interventions. Thus, potential confounders can impact the effectiveness of interventions, which makes studies like the ones by Geroe et al. [32] and Su et al. [29] especially important in the field of mental health in PALs, as they focus on vulnerable groups that might experience more stigmatisation. Nevertheless, it needs to be mentioned that psychosocial interventions are not always the most effective treatment option when it comes to mental disorders in leprosy patients. In severe cases, drug therapy might be the only way to alleviate symptoms [28,37].

When looking at India, the lack of available psychological interventions for PALs is very noticeable. While there have been numerous studies about the prevalence of psychological symptoms in PALs in India, there is no psychosocial support system in place despite single adopted policies aiming at the elimination of leprosy. However, as psychological disorders are often comorbidities of leprosy, it is important to implement a system for comprehensive psychological care [24,30,38,39].

When talking about the implementation of psychosocial interventions for PALs, based on this analysis, one has to keep in mind the different settings and countries the assessed programmes are functioning in. Besides a suitable psychosocial approach, it is important to have a legal foundation for these interventions to work. Thus, the concept of policy learning as described by Sanderson [40] could be one way to mitigate the problems arising when implementing new policies. This approach draws on present experiences from other countries to enhance the national policy regarding better psychosocial care for leprosy patients. This has the benefit of having an evidence-based alternative, while new programmes hold several uncertainties regarding financing, resource demand and appropriateness. Thereby, policymakers often do not look for new knowledge but for approaches that have been proven effective in other settings while addressing similar problems [40].

Yet, one needs to keep in mind that the success of these structural reforms highly depends on the society’s perception towards mental health and NTDs. The blaming of specific population groups, like PALs, can have an adverse effect on internalized as well as enacted stigma. This notion may be aggravated by apparent physical symptoms and deformities caused by leprosy, leading to marginalization of this population group, placing them in the focus of discriminatory aggression [41]. While this paper analyzes psychosocial interventions to improve the QoL and mental wellbeing of PALs, it is crucial to assess what needs to be done to prevent the deterioration of a patient’s mental health in the first place. This includes early detection strategies to reduce the risk of physical disability due to disease progression. Therefore, preventive measures need to be taken before the health deteriorates and to restrict the emergence of stigma and its effect on mental health [2,18,20]. While it is important to change hindering legislation for more integrated mental healthcare, social stigma and marginalisation are produced by people’s attitudes and behaviour towards affected people. Thus, appropriate health education and information dissemination are of utmost importance to support national policies and recommendations by the WHO [33]. This would suggest that in the specific case of India, interventions that promote the mental wellbeing of PALs should mainly be of psychoeducational nature to reach a large number of individuals. Hence, interventions operating on an interpersonal or community level would be most suitable for the Indian context. These eligibility criteria suggest the implementation of psychoeducational interventions as these can be applied in group-session as well as on individual basis. As misinformation and persisting misbeliefs are considerable influences on people feeling stigmatised and hence, suffering from a reduced QoL and mental disorders, psychoeducational methods can countervail these effects (5). Furthermore, this type of intervention requires the least amount of resources as it reaches a large number of people at once and can be easily integrated in the treatment process [19,12]. However, India is still suffering a tremendous lack of mental healthcare personnel which is needed in order to implement any kind of psychosocial intervention as they demand trained personnel [6,32]. Thereby, a stronger inclusion of Accredited Social Health Activists (ASHA) which are engaged on the Indian community level could be an interesting approach to enhance psychosocial interventions for PALs [42]. Furthermore, the inclusion of peer and lay counselors can be useful in the Indian setting, as it is a low-cost approach that achieves significant differences in people’s QoL and psychological health [38]. This could be connected with innovative technological-based interventions which still need to be proven effective in this setting.

Providing meaningful interventions for leprosy affected individuals and thus, improving their QoL highly contributes to the achievement of SDGs 3.3 and 3.4. By continuously educating people on transmission and symptoms of leprosy, early detection and hence, decreasing cases may be the result contributing to sub target 3.3. Furthermore, the implementation of effective psychosocial interventions supports the promotion of the mental wellbeing of leprosy patients, which may lead to a reduced premature mortality in this population group [43].

## 5 Limitations

One of the main limitations of this review was the limited availability of literature on this topic. This applies not only to the supportive literature, which is rather old, but especially to suitable studies that evaluate a possible psychosocial intervention to improve psychological wellbeing of leprosy participants. Moreover, the included studies show a wide heterogeneity in the used methodology. This may have impacted the comparability of the findings, however, since the term psychosocial interventions comprise a large range of possible approaches this heterogeneity was inevitable. As explained before, the different methodologies were necessary as they provide different perspectives on this topic [38]

## 6 Conclusion

Due to the obvious research gap in the field of leprosy and mental disorders, no systematic review has yet been done to consolidate the evidence regarding existing psychosocial interventions that address mental illnesses and a reduced QoL in leprosy patients, which makes this review the first of its kind. Thus, this paper aims at providing urgently needed knowledge to ensure that respective stakeholders can create effective treatment plans for mentally affected leprosy patients in low-resource settings such as India.

A leprosy diagnosis is often described as a life-altering event. Relationships and activities, people have previously engaged in, have to be modified or cannot be continued. Yet, the diagnosis does not imply certainty about treatment and future perspectives. By equipping mental health and leprosy advocates with evidence-based research, extensive change can be achieved by making overdue decisions towards a better treatment provision for PALs possible. Overall, the findings of this paper will help leprosy advocates to aim for better research in the relationship between mental health and leprosy. They will further drive awareness to the urgent need of change within the Indian stigma policies and thus, will lead to an improved understanding about leprosy among society. Helping communities to change their perspectives towards the disease will have a major impact on discrimination and stigmatisation against PALs [44]. Consequently, this attitude transformation will aid the primary outcome of this paper, the assessment of effective psychosocial interventions to improve the psychological wellbeing of leprosy patients and how it can be applied to the Indian setting. This review contributes to improved practices within the mental health care in India, as it provides urgently needed data for future implementations.

## 7 Future recommendations

Keeping in mind the findings of this systematic review, it is important to formulate reasonable policy and research recommendations to facilitate the current situation in not only India but also in other endemic countries. Therefore, it is crucial to increase research towards effective, especially cost-effective psychosocial interventions that help to promote the mental wellbeing of leprosy patients. Furthermore, already available interventions need to be proven for their long-term effectiveness, such as the studies included in this review, and if needed adapted accordingly. The introduced approaches further need to be tested on different patient groups with varying demographics to be able to adapt the interventions to certain population groups if required. These suggestions should be followed by leprosy advocates in future research to provide more detailed data on the interventions and their qualities.

Additionally, it is of utmost importance to improve educational services for the overall population in endemic countries to reduce fear, misinformation, and stigmatisation towards the disease as well as mental disorders that might be connected to it and those affected by it. This can further help to improve psychiatric care in India, since the low number of psychiatric centres limits the educational options available. As a result of persisting stigma, psychiatry is still not appropriately represented in the medical training programmes in India [45]. These recommendations are also applicable to other endemic countries lacking mental health services for PALs. However, national regulations differ, wherefore this review can serve as a baseline guideline to emphasise the benefits of effective psychosocial interventions and their effectiveness.

Furthermore, it is important to emphasise the impact of technology-based and other novel interventions, whereby some have been introduced here. However, other approaches that have been proven effective in managing depression and anxiety, such as art-based therapy or storytelling, could be a low-resource alternative to reach more PALs [35,36]. Digitalisation brings more advantages as apps and internet-based tools can further increase access to reach people who might live too far from medical centres [46]. Overall, it is important to consider innovative approaches that have been proven effective in similar settings, as they could be a useful asset in improving the mental wellbeing and QoL of PALs.

## Supporting information

S1 Checklist SI 6 PRISMA checklist. (DOCX)
